# Linking CRISPR–Cas9 double-strand break profiles to gene editing precision with BreakTag

**DOI:** 10.1038/s41587-024-02238-8

**Published:** 2024-05-13

**Authors:** Gabriel M. C. Longo, Sergi Sayols, Andriana G. Kotini, Sabine Heinen, Martin M. Möckel, Petra Beli, Vassilis Roukos

**Affiliations:** 1 Institute of Molecular Biology (IMB), Mainz, Germany; 2 Department of Biology, Medical School, University of Patras, Patras, Greece; 3 Johannes Gutenberg University (JGU), Mainz, Germany

**Keywords:** Genomics, Targeted gene repair

## Abstract

Cas9 can cleave DNA in both blunt and staggered configurations, resulting in distinct editing outcomes, but what dictates the type of Cas9 incisions is largely unknown. In this study, we developed BreakTag, a versatile method for profiling Cas9-induced DNA double-strand breaks (DSBs) and identifying the determinants of Cas9 incisions. Overall, we assessed cleavage by SpCas9 at more than 150,000 endogenous on-target and off-target sites targeted by approximately 3,500 single guide RNAs. We found that approximately 35% of SpCas9 DSBs are staggered, and the type of incision is influenced by DNA:gRNA complementarity and the use of engineered Cas9 variants. A machine learning model shows that Cas9 incision is dependent on the protospacer sequence and that human genetic variation impacts the configuration of Cas9 cuts and the DSB repair outcome. Matched datasets of Cas9 and engineered variant incisions with repair outcomes show that Cas9-mediated staggered breaks are linked with precise, templated and predictable single-nucleotide insertions, demonstrating that a scission-based gRNA design can be used to correct clinically relevant pathogenic single-nucleotide deletions.

## Abstract (web summary)

The genome-wide landscape of Cas-induced double-strand breaks and end structures is profiled at nucleotide resolution.

## Main

CRISPR–Cas9 has revolutionized genome editing in both basic and applied biomedical research as a means toward programmable, targeted and precise correction of genetic diseases^1–4^. Although the DNA-targeting specificity of CRISPR–Cas9 has been enhanced by redesigning guide RNAs (gRNAs) and engineering variants with higher fidelity, Cas9 template-free editing in eukaryotic cells has not yet been controlled at the required level for high-precision use in therapeutic applications^5^.

Cas9-mediated DNA editing was initially thought to result in random insertions and deletions (indels); however, mounting evidence indicates that the repair of Cas9-induced DNA breaks is not random but, rather, is strongly dependent on the sequence context of the target site^6–9^. Large datasets coupling CRISPR–Cas9 target sequences with their respective editing results have been used to develop models for predicting repair outcomes in mammalian cells^9–13^. Despite this progress, it is still unclear how Cas9 target sequences mechanistically influence DNA repair outcomes. One possible scenario is that different types of Cas9 incisions are associated with distinct editing outcomes, as shown in individual cases of staggered Cas9-mediated DNA double-strand breaks (DSBs) linked to single-nucleotide insertions^14–17^. Although it is now well accepted that Cas9 can cleave DNA in both blunt and staggered configurations^14–16,18^, where, how and at what frequencies these alternative DSB end structures are formed remains unknown. Moreover, the impact of genetic variation on Cas9 scission and editing outcomes has not been investigated—an important gap in knowledge as CRISPR-based therapeutics become increasingly achievable. The scarcity of systematic information on the outcome of Cas9 nuclease function can be attributed mainly to the lack of scalable tools that can simultaneously measure the frequency, location and structure of Cas9-induced DNA breaks.

To address this issue, we developed a next-generation sequencing (NGS)-based methodology, called BreakTag, to comprehensively profile the genome-wide DSB landscape of Cas nucleases along with their end structures at nucleotide resolution. Using BreakTag, we characterized the Cas9 scission at a total dataset of approximately 150,000 endogenous loci targeted by approximately 3,500 single guide RNAs (sgRNAs), and we identified determinants of Cas9 incisions. Furthermore, we investigated the impact of human genetic variation on Cas9 scission profile, and we identified Cas9 variants with biases in cleavage configuration and alternate sequence determinants. Finally, we devised a machine learning model to survey pathogenic single-nucleotide deletions that can be corrected by exploring sequence determinants of staggered cleavage and the predictability of insertions. Our findings establish that the predictability and precision of Cas9-mediated genome editing is mechanistically linked to the Cas9 incision structure and suggest that the flexible cut profile of Cas9, along with engineered nuclease variants with skewed scission profiles, can be harnessed for precise and personalized indel engineering.

## BreakTag systematically profiles genome-wide Cas9 activity

**Fig. 1:**
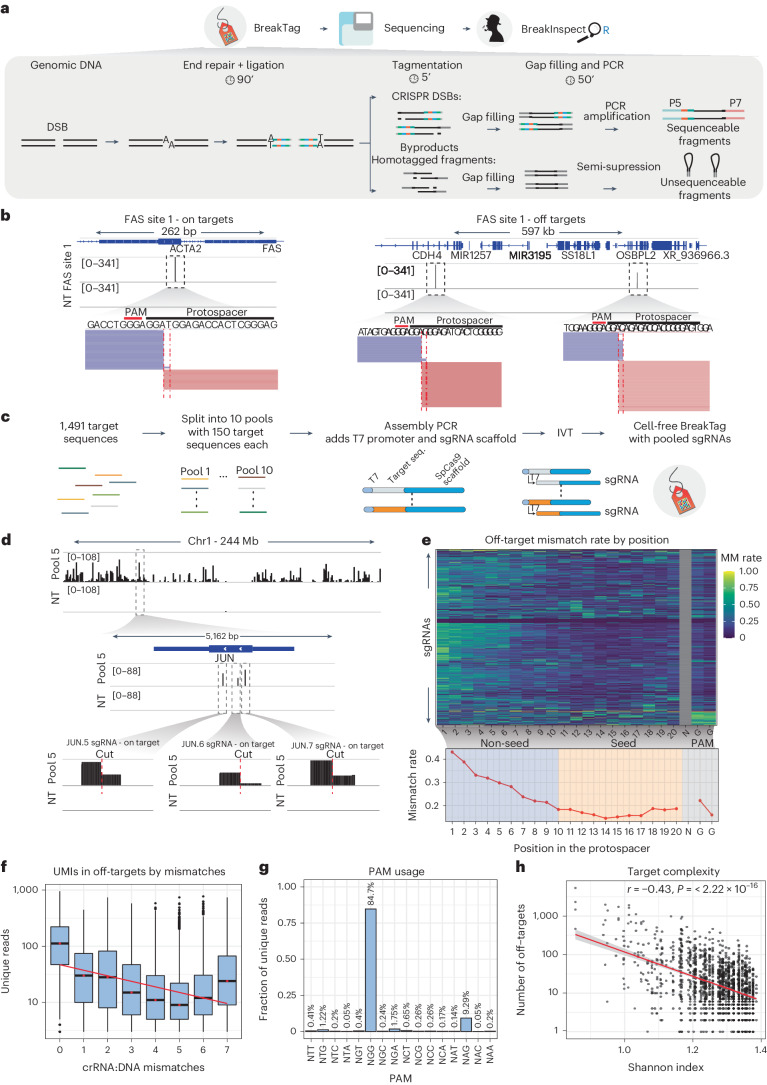
BreakTag profiles CRISPR on-target and off-target DSBs. **a**, Scheme depicting the experimental workflow for BreakTag (Supplementary Note 1). **b**, Representative IGV snapshot showing processed BreakTag data of the on-target DSB of the ‘FAS site 1’ gRNA (left) and two off-target sites (right). Zoomed-in views of the cut site (red dotted lines) and raw mapped reads (blue/pink rectangles) are shown below. NT, non-target control. gDNA from U2OS cells was used. **c**, HiPlex BreakTag strategy. Previously reported genomic Cas9 target sequences (ref. ^7^) were bioinformatically split into 10 pools, each containing approximately 150 sequences. A T7 promoter sequence was added to the 5′ end of each sgRNA protospacer, and a Cas9 sgRNA scaffold sequence was added at the 3′ end by a PCR assembly reaction, which generates a dsDNA template for T7 IVT. T7-transcribed sgRNAs were used for BreakTag with Cas9 in gDNA from HepG2 cells. **d**, IGV snapshot of chromosome 1, depicting cleaved sites for Pool 5 of the HiPlex1 dataset. Zoomed-in views of on-target DSBs of sgRNAs targeting the JUN gene are shown below. **e**, Top, heatmap depicting crRNA:DNA mismatch accumulation along the protospacer of 92,375 off-target sites identified by BreakTag on 1,418 sgRNAs in the HiPlex 1 dataset. Bottom, plot of the average mismatch rate along the protospacer. **f**, Number of unique reads after de-duplication using UMIs for identified target sites containing 0–7 crRNA:DNA mismatches. *n* = 92,375 cleaved sites (*n* = 84,104 independent cleaved on-target/off-target sites). Boxes characterize the sample using the lower quartile (Q1), median quartile (Q2) and upper quartile (Q3) and the interquartile range (IQR = Q3−Q1), and whiskers extend to the most extreme data point that is no more than 1.5× IQR from the edge of the box. The red line depicts the best fit of a linear model relating BreakTag reads in target sites to mismatches. **g**, Percentage of unique reads for identified target sites containing non-canonical PAM sequences. **h**, Correlation between the number of measured off-target cutting events and sequence complexity of the target site measured according to the Shannon index. IGV, Integrative Genome Viewer; MM, mismatch. Source data

**Extended Data Fig. 1:**
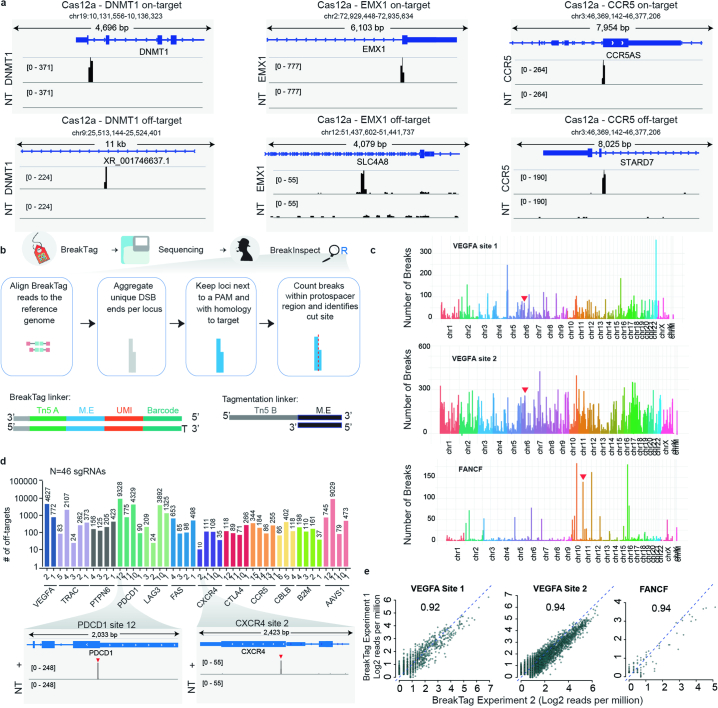
BreakTag and BreakInspector allow high-throughput, genome-wide assessment of Cas9 and Cas12a on- and off-targets. a, IGV snapshots of Cas12a on targets and representative off-targets of 3 gRNAs. b, Schematics of BreakInspectoR analysis workflow. c, Manhattan plots showing off-targets nominated for ‘VEGFA site 1’, ‘VEGFA site 2’ and ‘FANCF’. Red arrowheads indicate on-target sequences. BreakTag was performed in gDNA from U2OS cells. d, Number of off-targets mapped by BreakTag in gDNA of U2OS cells digested with Cas9 and 46 different clinically relevant gRNAs^19^. Representative IGV snapshots of the on-target region of ‘PDCD1 site 12’ and ‘CXCR4 site 2’ are shown below. Off-targets were called using a low threshold of at least 3 reads and up to 7 mismatches. e, Correlation between two independent BreakTag runs for three sgRNAs commonly used in the benchmarking of off-target-nominating tools. Source data

To characterize and identify the determinants of the Cas9 scission profile, we developed BreakTag, an efficient method for unbiased, high-throughput and systematic profiling of Cas9-mediated DSBs. BreakTag is a highly scalable protocol that maps free DSB ends in genomic DNA (gDNA) digested by ribonucleoproteins (RNPs) in vitro in four simple steps: (1) an end repair/A-tailing step prepares the ends for (2) ligation with an adaptor with a unique molecular identifier (UMI) for DSB count and a sample barcode for sample multiplexing, followed by (3) tagmentation with Tn5 transposase and (4) polymerase chain reaction (PCR) amplification of ligated fragments (Fig. 1a and Methods). The DSB enrichment step occurs during PCR, yielding a fast (<6 h for ready-to-sequence libraries), highly scalable and cost-efficient method for mapping CRISPR nuclease DSBs genome wide. DSB reads start at the cut site, and read directionality is preserved with each side of the break mapping to opposite strands (Fig. 1b). Moreover, the end repair step in our experimental procedure enables the enrichment of DSBs containing single-stranded DNA (ssDNA) overhangs, allowing off-target nomination of staggered-cleaving nucleases such as Cas12a with the same protocol (Extended Data Fig. 1a). We partner BreakTag with BreakInspectoR, a bioinformatics pipeline for identifying and counting Cas9-induced DSBs in BreakTag data (Extended Data Fig. 1b,c; see Data, Materials and Code availability sections for links to the code).

**Extended Data Fig. 2:**
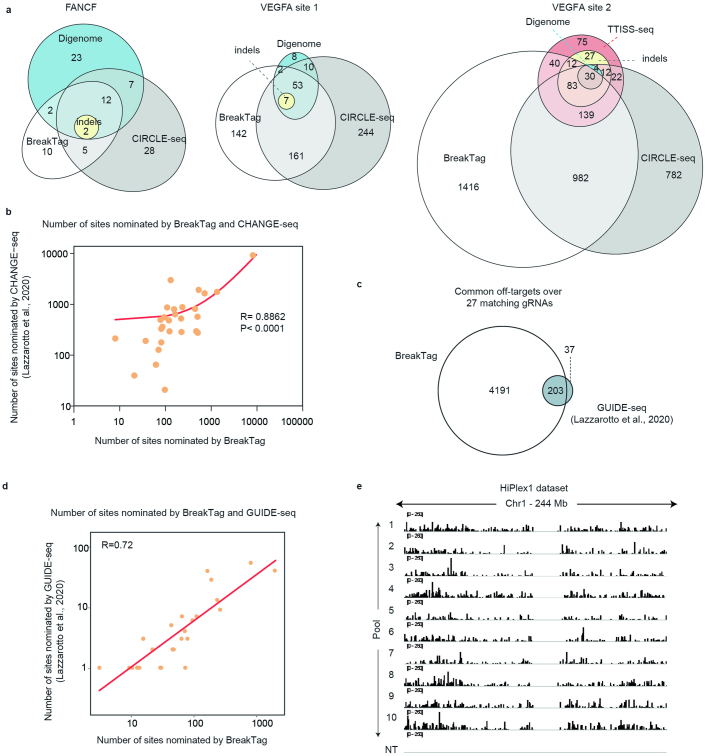
Benchmarking of BreakTag against other off-target nominating tools. a, Venn diagrams showing the overlap between sites nominated by BreakTag, DIGENOME-seq^20^, and CIRCLE-seq^21^. Off-targets were selected for validation using targeted deep sequencing. TTISS-seq^37^ was used to generate a refined list of *in cellulo* VEGFA site 2 off-targets due to its high promiscuity. A minimum of 8 reads and a maximum of 6 mismatches was used for BreakTag off-targets in order to match public available data’s thresholds. b, Correlation between number of off-targets nominated by CHANGE-seq^19^ and BreakTag over 44 gRNAs arbitrarily selected from the CHANGE-seq dataset. c, Common off-target sites identified by GUIDE-seq (data produced in^19^) and BreakTag over matching 27 gRNAs. For GUIDE-seq only targets supported by at least 8 reads, up to 6 mismatches between crRNA:DNA and an NGG PAM were considered; for BreakTag targets supported by at least 8 reads, up to 6 mismatches and a FDR < 1% were considered. d, Correlation between the number of off-targets nominated by BreakTag and GUIDE-seq data^19^. e, IGV snapshot of chromosome 1 of HepG2 cells digested with Pools 1–10 from the HiPlex1 library. Each bar represents a cleaved site. NT: nontarget control. Source data

To benchmark BreakTag against previously developed tools, we profiled the off-target landscape of 46 sgRNAs^19^ (Supplementary Table 1) targeting 12 clinically relevant genes with *Streptococcus pyogenes* (SpCas9, hereafter ‘Cas9’). We observed a wide range of off-targets, identifying sgRNAs with either high specificity or promiscuity (for example, CXCR4 site 2: 10 off-targets; PDCD1 site 12: 9,328 off-targets) (Extended Data Fig. 1d and Supplementary Table 2). Of note, BreakTag showed excellent reproducibility across different gRNAs commonly used to benchmark off-target mapping tools (Extended Data Fig. 1e). To benchmark BreakTag, we compared the lists of off-targets nominated by DIGENOME-seq^20^ and CIRCLE-seq^21^. BreakTag identified previously characterized off-targets but also sites that were absent in DIGENOME-seq and CIRCLE-seq datasets (Extended Data Fig. 2a). Furthermore, we identified an excellent correlation between the number of sites nominated by BreakTag and CHANGE-seq, an improved version of CIRCLE-seq (Pearson *r* = 0.8862, *P* < 0.0001) (Extended Data Fig. 2b). We performed targeted deep sequencing of off-targets nominated by DIGENOME-seq, CIRCLE-seq and BreakTag to validate bona fide Cas9 unintended mutations, and we observed that most sites that showed editing were nominated by all three methods (Extended Data Fig. 2a). We next tested BreakTag against GUIDE-seq, a sensitive in cellulo method that relies on the incorporation of double-stranded DNA (dsDNA) donor tags at the cut site^22^ over 27 matching gRNAs^19^. We observed a complete overlap of off-targets nominated with BreakTag and GUIDE-seq in 19 out of 27 tested gRNAs (Extended Data Fig. 2c). Approximately 85% of all targets nominated by GUIDE-seq were also nominated by BreakTag across all tested gRNAs (Extended Data Fig. 2c). Of note, we observed an excellent correlation between the number of off-targets nominated per gRNA for the tested methods (*r* = 0.72) (Extended Data Fig. 2d).

To further investigate the determinants of CRISPR–Cas9 off-target activity, we used the scalability of BreakTag to develop HiPlex BreakTag, which takes advantage of high-throughput enzymatic sgRNA synthesis and the pooling of several reactions. We split 1,491 previously described sgRNA sequences targeting human genes (hereafter referred to as the ‘HiPlex1’ library)^7^ into 10 pools (~150 sequences per pool) (Supplementary Table 1) and produced them by T7-mediated in vitro transcription (IVT) (Fig. 1c). BreakTag was then performed using as input gDNA digested with the various sgRNA pools. This procedure identified 92,375 on-targets/off-targets (1,418 of the 1,491 on-target sites were cut) (Supplementary Table 3), validating the efficacy of our approach (Fig. 1d and Extended Data Fig. 2e). We used this dataset to investigate the positional effects of incorrect base pairing (mismatches) between the CRISPR RNA (crRNA) and target DNA, complementing previous findings^18,19^. We observed that protospacer-adjacent motif (PAM)-distal regions were more permissive to incorrect base pairing than the PAM-proximal portion of the protospacer (Fig. 1e). In accordance with previous observations showing that mismatches within the seed sequence disrupt R-loop formation and ablate DNA cleavage^23,24^, target cleavage frequency was inversely correlated with the number of mismatches (Fig. 1f)^19^. Previous reports showed that Cas9 can use alternative PAM sequences^18,19^. We identified that 84.7% of the cleaved sites were found next to the canonical PAM NGG, followed by NAG (9.29%) and NGA (1.75%), showing that non-canonical PAMs are used, albeit with lower frequency (Fig. 1g). We further identified an inverse correlation between the number of off-targets and the sequence target complexity (measured by the Shannon index; *r* = −0.43, *P* < 2 × 10^−16^) (Fig. 1h), suggesting that a selection of more complex target sites could be used as a strategy to minimize off-target activity. Taking these findings together, we conclude that BreakTag is a sensitive, fast and scalable methodology for detecting CRISPR–Cas9-induced DSBs and is proficient at identifying the determinants of off-target activity, thus complementing previous efforts^18,19^.

## BreakTag reveals the flexible Cas9 scission profile

**Fig. 2:**
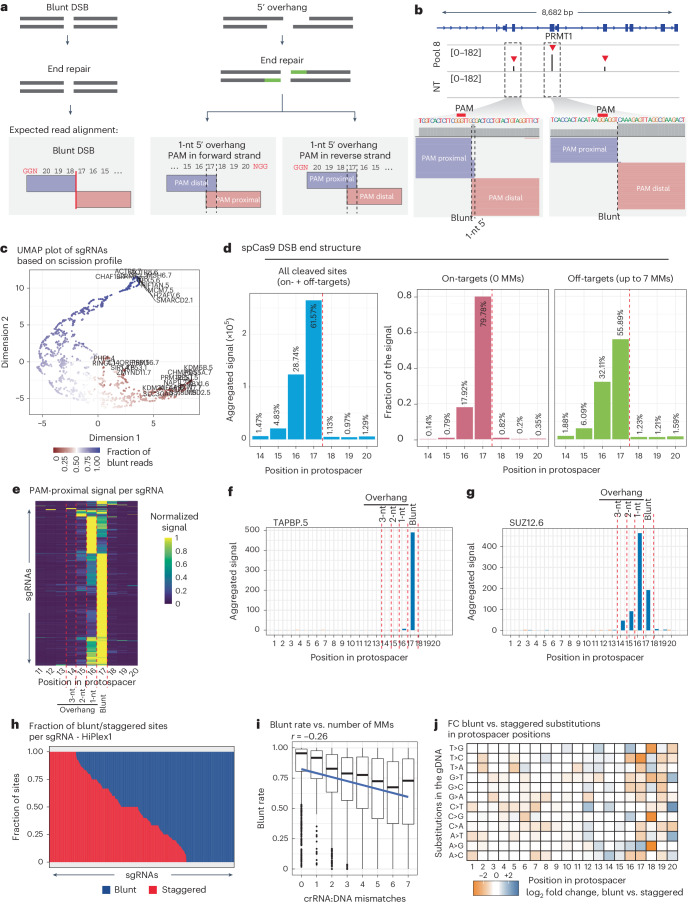
High-throughput analysis of SpCas9 scission profile. **a**, Schematic of read alignments for 5′ overhangs in BreakTag data. **b**, Representative IGV snapshot depicting three on-target DSBs identified by BreakTag. **c**, UMAP representation on two dimensions of relatedness between sgRNAs based on average scission profile. Dimensions 1 and 2 are representations in a reduced dimensional space (arbitrary units) of the scission profile. Color scale represents the fraction of signal at the expected cut site, ranging from 100% (blue) to 0% (red). **d**, Aggregated signal of different DSB end structures for all targets or grouped into NGG on-targets/off-targets in the HiPlex1 dataset. Position 17: blunt DSBs; 16–14: 5′ overhangs. The dotted line indicates the expected cut site for a blunt DSB. **e**, Accumulation of reads mapped onto the PAM-proximal strand (scaled) along the protospacer over 1,418 sgRNAs of the HiPlex1 dataset for all identified NGG targets. 17: blunt DSBs; 16–14: 5′ overhangs **f**,**g**, Examples of target sites at which Cas9 cuts preferentially in blunt or staggered configuration. Aggregated BreakTag signal along the protospacer for ‘TAPB.5’ sgRNA on-target and off-target (*n* = 3) (**f**). Aggregated BreakTag signal along the protospacer for ‘SUZ12.6’ sgRNA on-target and off-target (*n* = 56) (**g**). **h**, Columns represent the fraction of blunt (blue) or staggered (red) reads for on-targets/off-targets of a given sgRNA. **i**, Box plots showing the average blunt rate for sites containing up to seven crRNA:DNA mismatches. *n* = 26,802 sites with at least 16 reads in the PAM-proximal side. Boxes characterize the sample using the lower quartile (Q1), median quartile (Q2) and upper quartile (Q3) and the interquartile range (IQR = Q3−Q1), and whiskers extend to the most extreme data point that is no more than 1.5× IQR from the edge of the box. The blue line depicts the best fit of a linear model relating blunt rate in target sites to mismatches (Pearson *r* = −0.26, *P* < 2.2 × 10^−16^; *n* = 26,802 independent Cas9 on-targets/off-targets). **j**, Heatmap showing the log_2_ fold change of frequency of nucleotide substitutions along the protospacer in predominantly blunt sites (blunt raw reads > 66%) compared to predominantly staggered sites (blunt raw reads < 33%) (*n* = 26,802 sites with at least 16 reads in the PAM-proximal side). IGV, Integrative Genome Viewer; MM, mismatch; NT, non-target control. Source data

**Extended Data Fig. 3:**
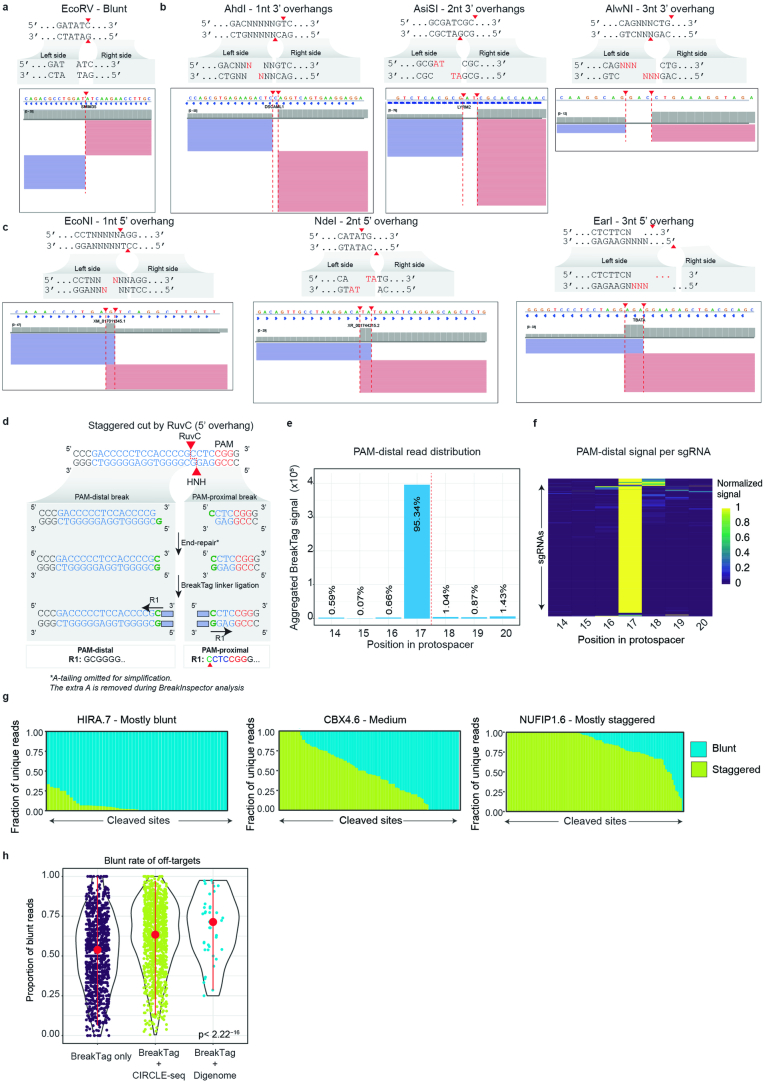
BreakTag allows profiling of Cas9 scission. a, gDNA of HEK293 cells was *in vitro* digested with a panel of restriction enzymes that generate blunt DSBs, b, 1–3 nt long 3’ ssDNA overhangs, or c, 1–3 nt long 5’ ssDNA overhangs at the cut site, and BreakTag was performed. IGV snapshots show raw mapped reads for a representative target site for each enzyme. Arrowheads indicate the start of DSB reads. d, Scheme depicting a staggered DSB with a 1 nt 5’ overhang. PAM-proximal side of the break starts 1 nt upstream (16|17) of the expected site for a blunt cut. e, Read distribution of the PAM-distal read along the protospacer. Because of the direction of the reaction to fill-in 5’ overhangs during end repair, PAM-distal reads map to position 17 (cut site from the HNH domain) for both blunt and staggered reads. f, PAM-distal signal distribution along the protospacer for each sgRNA used in the HiPlex 1 data set. g, Fraction of BreakTag reads accumulating on position 17 (blue), suggestive of a blunt incision, or in other positions of the protospacer (green), indicative of a staggered cut, for three sgRNAs. Each column represents a cleaved site including on and off-targets. h, Blunt rate of off-targets nominated exclusively by BreakTag or shared with CIRCLE-seq or Digenome-seq. The line range in red characterizes the sample using the median (Q2) - depicted with a point - and the range between percentiles 0.025 and 0.975 (n = 4,375 sites, two-sided ANOVA test comparing means, P-value < 2.22e-16). Source data

A unique advantage of BreakTag is that it allows the original DSB end structure to be retraced, as the filling-in of 5′ overhangs and removal of 3′ overhangs during BreakTag sample preparation should shift the expected start of the DSB reads, yielding a footprint of the original DSB end structure. To confirm this, we performed BreakTag on gDNA of cells in vitro digested with a panel of restriction enzymes having different cutting structures, and we assessed the read signatures around the expected cut site. We observed that blunt DSBs generated reads that abutted at the expected cut site (Extended Data Fig. 3a), whereas the use of restriction enzymes that generate 3′ or 5′ overhangs led to a clear gap or overlap between the DSB reads, respectively, with size corresponding to the length of the expected overhang (Extended Data Fig. 3b,c). We reasoned that applying the same rationale would enable an investigation of the scission profile of Cas9-induced DSBs. The RuvC domain of Cas9 can cleave the non-target strand at non-canonical positions, generating ssDNA 5′ overhangs^3,14–16,18^. In the scenario of a blunt DSB, both the RuvC and HNH domains cleave the DNA strands between the third and fourth nucleotide upstream of the PAM sequence (positions 18 and 17 of the protospacer, respectively), generating abutting DSB reads aligned at the expected cut site for blunt cuts (Fig. 2a and Extended Data Fig. 3d). If the RuvC domain cleaves the non-target strand upstream of the HNH domain, 5′ ssDNA overhangs are generated, and, upon end repair during BreakTag, the PAM-proximal and PAM-distal reads overlap and no longer abut (Fig. 2a,b and Extended Data Fig. 3d). We used this feature of BreakTag to assess the frequency of the different DSB end structures generated by Cas9. To this end, we used a subset of the HiPlex1 dataset with sites containing an NGG PAM, and at least 16 reads at the PAM-proximal side of the DSB, yielding a total of 38,141 on-target/off-target sites. Because the fill-in reaction occurs toward the PAM, the PAM-distal side of the break is expected to map between target positions 17 and 18 regardless of the RuvC cleavage position on the non-target strand (Extended Data Fig. 3e,f). Therefore, we extended BreakInspectoR to also parse the reads of each DSB into PAM proximal or PAM distal, and we used this feature to calculate the ‘blunt rate’, defined as the abundance of blunt DSBs profiled at the expected site for a blunt cut (between positions 17 and 18) relative to the total DSBs profiled in a region around [−3, +3] the expected cut site for the PAM-proximal read (Methods). The different sgRNAs self-organized based on their scission profile and preferred overhang length in the expected classes (Fig. 2c). Profiling the structure of Cas9-induced DSBs revealed that Cas9 preferentially generates blunt DSBs (61.57%), but a significant portion contains 5′ ssDNA overhangs (35.04%) (Fig. 2d, left). Interestingly, the presence of mismatches between the crRNA and gDNA influenced the Cas9 scission profile. In the absence of mismatches, 79.78% of the Cas9 DSBs were blunt, whereas approximately 18% of Cas9 DSBs were staggered (Fig. 2d, middle). At off-targets, the number of blunt breaks decreased (to 55.89%), whereas the percentage of staggered breaks increased (to ~40%) (Fig. 2d, right). The scission profile was target sequence dependent (Fig. 2e), with gRNAs showing nearly completely blunt Cas9 breaks (for example, TAPBP.5) (Fig. 2f) and others exhibiting a broader range of Cas9 cuts (for example, SUZ12.6) (Fig. 2g). The fraction of blunt/staggered breaks across their target sites was sgRNA dependent. In 15.07% of the sgRNAs tested, Cas9 cut almost exclusively in a blunt configuration (blunt reads > 90%), whereas, in 11.77%, Cas9 cut almost exclusively in a staggered fashion (staggered reads > 90%) (Fig. 2h and Extended Data Fig. 3g).

In line with our findings indicating that the target sequence and the presence of mismatches influence the Cas9 scission profile, we found that Cas9 blunt rate inversely correlates with the number of identified mismatches (Fig. 2i), suggesting that partial complementarity between the crRNA and target site favors more staggered Cas9 cuts. Changes in the blunt rate were higher if mismatches were located at positions 16–20 of the protospacer/target sequence, suggesting that these positions might be important for determining the profile of Cas9 scission (Fig. 2j). Given the unique ability of BreakTag to probe the end structure of Cas9 target-dependent proportion of blunt to staggered cuts, we investigated the blunt rate of off-targets nominated by BreakTag alone or shared with CIRCLE-seq and DIGENOME-seq. We observed that BreakTag-exclusive sites showed a higher proportion of staggered reads, suggesting that the end repair step might be beneficial to capture sites with a high proportion of staggered cuts (Extended Data Fig. 3h).

## Determinants of Cas9 scission profile mediate precise and predictable indels

**Fig. 3:**
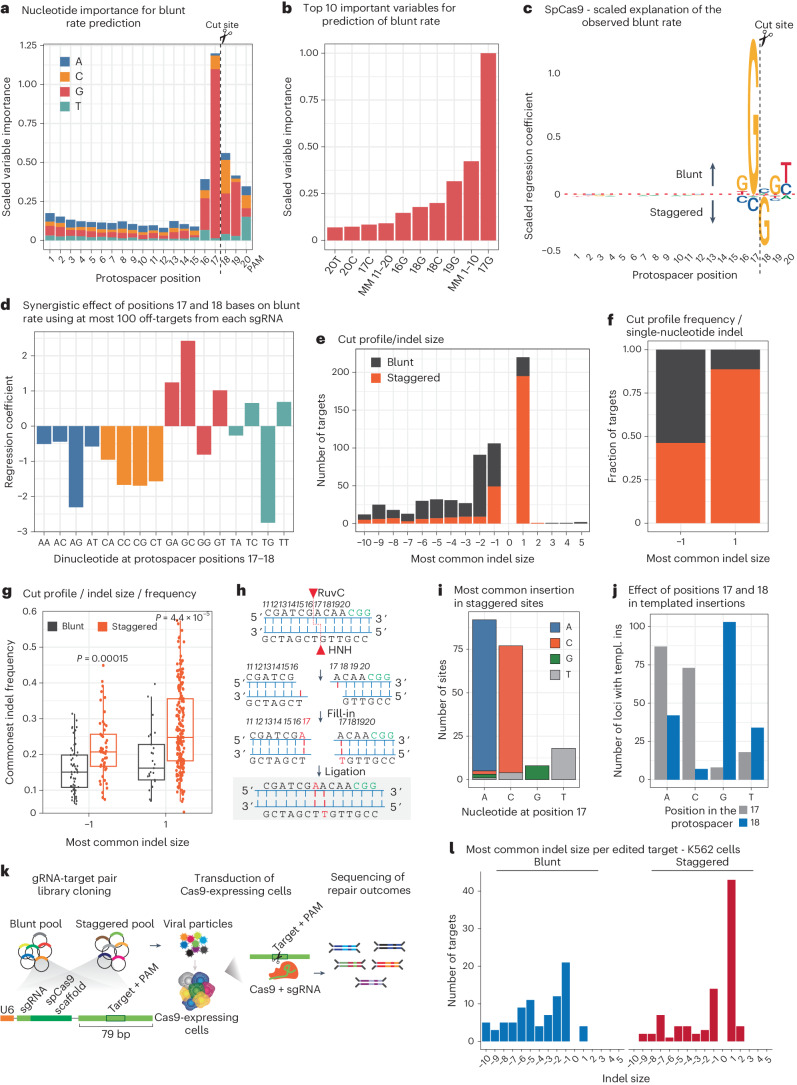
Sequence determinants of Cas9 scission profile. **a**, Importance of the nucleotide composition and position in the protospacer estimated by XGBoost. Values on the *y* axis are scaled to the most important nucleotide + position. **b**, Top 10 most important variables for the prediction of blunt rate. MM 1–10, mismatches in positions 1–10; MM 11–20, mismatches in positions 11–20. **c**, Observed blunt rate explained by the sequence composition of the protospacer. Coefficients of a linear regression model fit to the nucleotide composition independently on each position of the protospacer are shown as letters scaled according to the importance of that nucleotide and position. **d**, The effect of all possible nucleotide combinations in position 17 and 18 in the blunt rate prediction. **e**, HiPlex dataset 2 was performed to assess the scission of 610 sites in a matched dataset with known repair outcomes (+1-nt to +5-nt insertions, −1-nt to −10-nt deletions)^10^. An equal number of blunt and staggered breaks sites were used for the analysis (*n* = 610). **f**, Cut profile frequency in single-nucleotide indels (two-tailed Fisher’s test: odds ratio = 8.99, *P* = 8.345 × 10^−16^). Colors represent the fraction of blunt (gray) or staggered (orange) sites showing single-nucleotide indels. **g**, Frequency of 1-nt deletions or insertions in relation to scission profile (two-sided *t*-test: *P* = 0.00015 for −1 deletions, *P* = 4.4 × 10^−5^ for +1 insertions). *n* = 1,326 Cas9 sites. Box plots show the lower quartile (Q1), median quartile (Q2) and upper quartile (Q3), with whiskers extending up to 1.5× the interquartile range (IQR = Q3−Q1) from the box edges. **h**, Scheme depicting how 1-nt 5′ overhangs lead to templated insertions. **i**, Most common insertion at staggered sites according to nucleotide at position 17. **j**, Number of loci with templated insertion according to the base composition at positions 17 (gray) or 18 (blue). **k**, Schematics of gRNA-target pair experimental design for the blunt and staggered pools. **l**, Most common indel size found per edited target in K562–Cas9 cells. A total of 199 gRNA-target pairs (93 staggered and 106 blunt) were used for this analysis after filtering for sites with at least 100 mutated reads and not detected in the negative control. templ. ins, templated insertions. Source data

**Extended Data Fig. 4:**
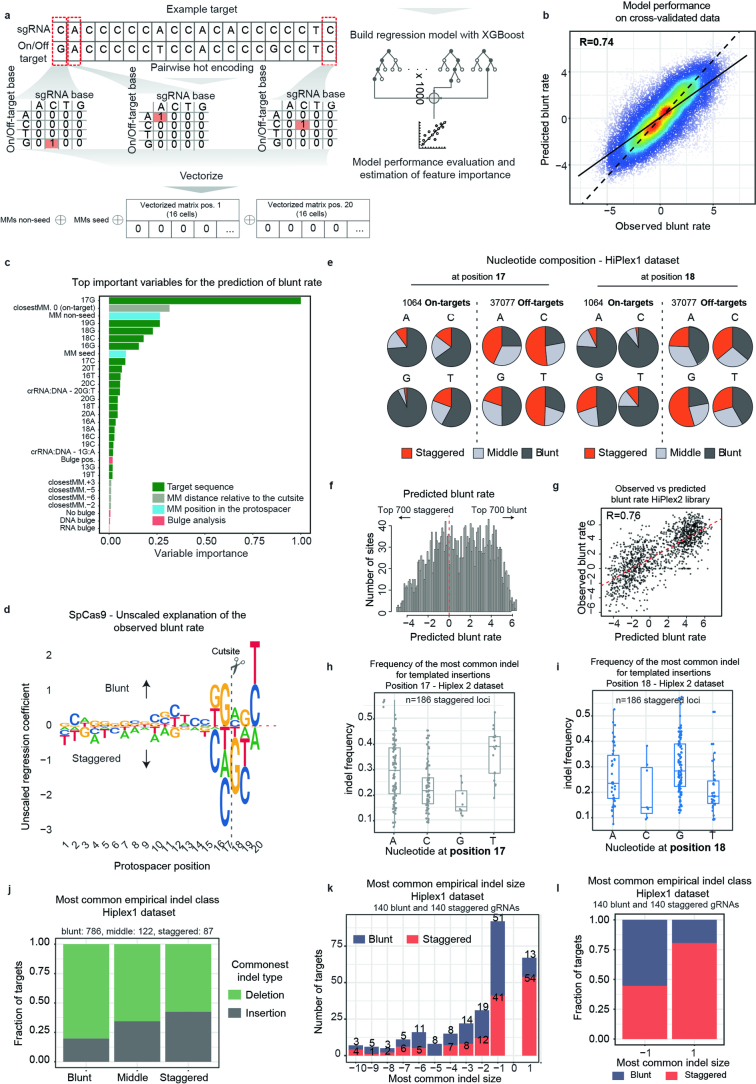
Determinants of Cas9 scission profile. **a**, Schematics of XGBoost method trained on BreakTag data. Training set consisted of a balanced set of 18,759 on and off-targets with a coverage of at least 16 reads in the PAM-proximal strand. **b**, Model performance evaluation using cross-validated data (Ten rounds of cross-validation). Panel shows the correspondence between expected (predicted) and observed log2 ratio of reads indicating a blunt or a staggered cut. **c**, Scaled feature importance estimated by XGBoost. **d**, Unscaled sequence explanation of the observed blunt rate using at most 100 off-targets identified by BreakTag for each sgRNA of the HiPlex1 library. **e**, The effect of each base at positions 17 (left) and 18 (right) in the scission profile for on and off-targets in the HiPlex1 library for sites with at least 16 reads in the PAM-proximal strand**. f**, Distribution of the predicted blunt rate for 2,791 gRNAs^10^. **g**, Correlation between predicted blunt rate by our model and observed blunt rate using BreakTag for top 700 staggered and top 700 blunt gRNAs identified. **h**, Frequency of the most common indel for templated insertions as a function of nucleotide at position 17 for all staggered-cleaved loci with a + 1 indel as the main repair outcome (*n* = 186). Box plots show the lower (Q1), median (Q2), and upper quartile (Q3), with whiskers extending up to 1.5 times the interquartile range (IQR = Q3 − Q1) from the box edges. **i**, Frequency of the most common indel for template insertions as a function of nucleotide at position 18 for 186 staggered loci with templated insertions. **j**, Fraction of targets where the most common repair outcome was a deletion (green) or insertion (gray). Cuts were grouped into ‘blunt’ (66-100% of blunt reads), ‘middle’ (33-66% of blunt reads) and ‘staggered’ (0-33% of blunt reads). Publicly available amplicon sequencing data was used^7^. **k**, Most common indel size as a function of scission profile. Cuts were grouped into ‘blunt’ (>=50% of blunt reads) and ‘staggered’ (<50% of blunt reads). **l**, Proportion of sites where the most common outcome was −1 (1nt deletion) or +1 (1nt insertion) as a function of scission profile. Source data

To identify important features influencing whether Cas9 cuts in blunt or staggered configuration, we trained an XGBoost regression model using the two-dimensional (2D) one-hot-encoded representation of the correspondence between the 20 nucleotides (nt) of the protospacer and guide sequences as predictors, together with the number of mismatches in the non-seed (positions 1–10) and seed (positions 11–20) parts of the protospacer. The blunt rate for the cleaved loci from our HiPlex1 library dataset was used as the target for this prediction (Extended Data Fig. 4a). Our model achieved high performance, as measured by the correlation between the predicted and observed blunt rates in the cross-validated sets (*r* = 0.74) (Extended Data Fig. 4b). The high predictive power of our model allowed us to investigate important positions within the protospacer that determines whether Cas9 cleaves the target DNA in a staggered or blunt manner. We observed that positions 16–20 (5 nt upstream of the PAM) were important for predicting the scission profile, with guanines at positions 17 and 18 having the highest importance (Fig. 3a,b and Extended Data Fig. 4c). We next sought to identify sequence compositions associated with a blunt or staggered cut by interrogating the importance of each base along the protospacer. Strikingly, we identified that a G at position 17 was predictive for a blunt DSB, whereas a G at position 18 was associated with staggered DSBs (Fig. 3c and Extended Data Fig. 4d).

To investigate the effects of 17G and 18G on Cas9 scission with our dataset, we grouped the cleaved sites into ‘blunt’ (0–33% of PAM-proximal reads mapping outside of position 17: staggered reads), ‘middle’ (33–66% staggered reads) and ‘staggered’ (66–100% staggered reads). Cas9 was, in general, more likely to cut blunt at on-target sequences than at off-targets where mismatches are present (ANOVA: *P* < 2 × 10^−16^) (Fig. 2d,i and Extended Data Fig. 4e). In accordance with the model predictions, Cas9 was more likely to cleave in a blunt configuration at sites with a G at position 17 compared to sites with A, C or T, at both on-targets and off-targets (Pearson’s chi-squared test: *P* < 2 × 10^−16^) (Extended Data Fig. 4e). In contrast, if a G occupied position 18, Cas9 was more likely to cleave in a staggered configuration than if A, C or T occupied that position (Pearson’s chi-squared test: *P* < 2 × 10^−16^) (Extended Data Fig. 4e). We further investigated the combination of nucleotides at positions 17 and 18 to determine their preference for either blunt or staggered cuts. Interestingly, the combination of 17T|18G had the most significant impact on promoting staggered cuts, whereas 17G|18C favored blunt breaks (Fig. 3d). We conclude that the base composition surrounding the DSB is a strong determinant of the Cas9 scission profile.

Previous evidence supported an association between Cas9 scission and repair outcome^14–16^, but the lack of scalable methods to assess scission profiles has precluded a systematic investigation. We deployed our machine learning model to 2,791 genomic gRNA targets, for which the repair outcome was previously characterized^10^, to predict the blunt rate for each gRNA sequence (Extended Data Fig. 4f). We then selected the predicted top 700 most blunt and top 700 most staggered sites for HiPlex BreakTag (hereafter referred to as the ‘HiPlex2’ library) to correlate their Cas9 scission profile with their empirical repair outcome (Supplementary Tables 1 and 4). The predicted blunt rate of this dataset was highly correlated with the actual scission profile obtained by BreakTag, confirming the robustness of our model (Extended Data Fig. 4g). When interrogating the scission profile as a function of the most common empirically observed indel size for each site, we observed that blunt cuts were equally represented across indel size (Fig. 3e). By contrast, a striking enrichment of staggered sites was found at genomic loci that are repaired as single-nucleotide insertions (+1 indels) (Fig. 3e). Over 90% of sites with a +1 indel as the most common repair outcome were staggered DSBs, demonstrating a clear association between scission profile and DNA repair (Fig. 3f). Staggered breaks generated more precise indels (that is, at a higher frequency) compared to blunt cuts for −1 and +1 indels (Fig. 3g). Precise insertions are desirable repair outcomes in the context of correcting pathogenic alleles and inducing gene knockouts. To understand the effect of sequence on the efficiency of templated insertions, we investigated the number of loci for which the most frequent repair was a templated insertion as a factor of base composition at positions 17 and 18 of the protospacer. If the ssDNA overhang at the cut site is used as a template for repair, we would expect that the most common insertion would be a copy of the overhang sequence. Because most overhangs generated by Cas9 are 1 nt long (Fig. 2d), we anticipated that position 17 would be duplicated in most cases (Fig. 3h). Indeed, the most common nucleotide inserted at staggered sites was a duplication of the base at position 17, indicating that template insertions are a common repair outcome of staggered DSBs (Fig. 3i). Target sites with G at position 17 showed a low number of templated insertions, as expected for blunt cuts (Fig. 3i,j and Extended Data Fig. 4h). By contrast, target sites with G in position 18 were more likely to use the nucleotide at position 17 as the template for the single-nucleotide insertions (Fig. 3j and Extended Data Fig. 4i), suggesting that target sequences with a specific nucleotide composition can be selected for precise, predictable and desirable genome editing.

We expanded our scission profile and indel analysis by investigating the most common indel outcome as a function of scission identity in our HiPlex1 dataset (generated in HepG2 gDNA), for which amplicon sequencing data are available^7^. Insertions were enriched at staggered-cleaved target sites compared to blunt (Extended Data Fig. 4j). In line with our previous findings, we observed that 1-nt insertions were highly associated with staggered DSBs (Extended Data Fig. 4k), with approximately 80% of 1-nt insertions being produced by staggered cuts (Extended Data Fig. 4l).

**Extended Data Fig. 5:**
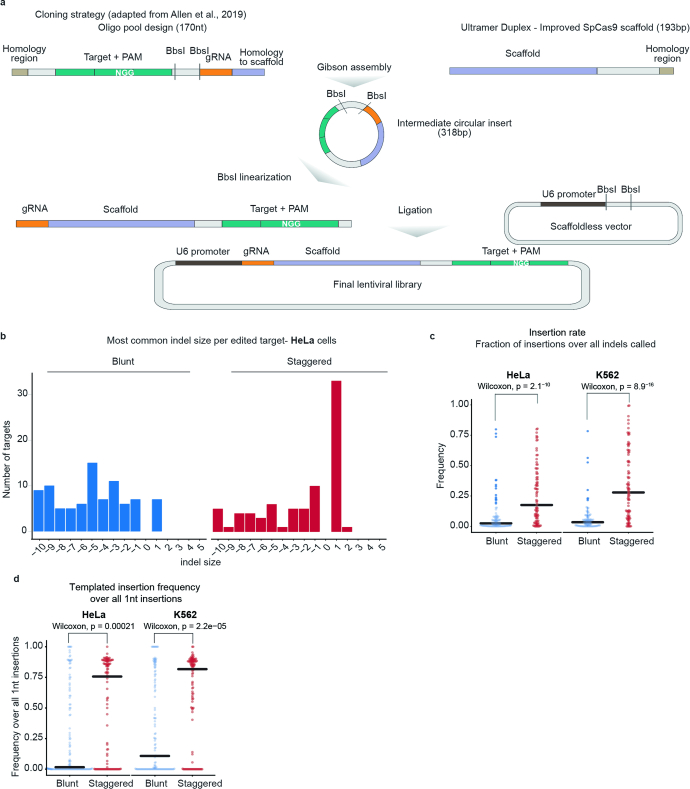
Parallel assessment of indel outcomes of target sequences predicted to be cut preferably in a blunt or staggered manner. **a**, Schematics of the strategy used to clone gRNA-target pairs into a lentiviral vector (adapted from^10^). Briefly, we designed the 79nt portion of the pathogenic allele carrying the deletion and PAM and its gRNA and ordered it in a Pool format. We performed a Gibson assembly reaction with an Ultramer Duplex containing a portion of the improved SpCas9 scaffold. The intermediate circular insert was linearized and ligated into a scaffoldless pKLV2-U6(BbsI)-PKGpuro2ABFP-W (addgene #67974). **b**, Most common indel size found per edited target in HeLa-Cas9. A total of 200 gRNA-target pairs (91 staggered and 109 blunt) were used for this analysis after filtering for sites with at least 100 mutated reads and not detected in the experiment performed with cells not expressing Cas9. **c**, Insertion rate of target sequences predicted to be cleaved preferably in a blunt or staggered manner. Insertion rate was calculated as the fraction of insertion over all indels called. Horizontal lines represent the median values. A two-sided Wilcoxon test was performed to assess the significance of the differences observed between the mean signed ranks of the two conditions being compared (HeLa blunt vs. HeLa staggered P-value 2.1e-10; K562 blunt vs. K562 staggered P-value 8.9e-16; n = 399 independent Cas9-induced cutsites). **d**, Frequency of templated insertions over all +1 indels. Insertions were considered as templated when the inserted base is the same nucleotide found in position 17 of the protospacer. Horizontal lines represent the median values. A two-sided Wilcoxon test was performed to assess the significance of the differences observed between the mean signed ranks of the two conditions being compared (HeLa blunt vs. HeLa staggered P-value 0.00021; K562 blunt vs. K562 staggered P-value 2.2e-5; n = 399 independent Cas9-induced cutsites). Source data

To further demonstrate that a pre-selection of target sites with predicted scission profile can be leveraged for increasing insertion precision, we tasked our machine learning trained on SpCas9 HiPlex BreakTag data to predict the blunt rate of Cas9 at various human target sequences, and we grouped them into ‘blunt’ and ‘staggered’ groups, showing the highest and lowest blunt rate, respectively (Supplementary Table 10). We then applied a gRNA-target pair cloning strategy^10^ to assess in parallel the repair outcome of sites predicted to be cut preferably in a blunt or staggered manner. In brief, we designed genomic cassettes of selected target sequences predicted to be cut in blunt or staggered configuration along with its targeting gRNA as pools cloned into lentiviral vectors (Fig. 3k and Extended Data Fig. 5a). Cas9-expressing K562 and HeLa cells were then transduced with the blunt or staggered pool; the gDNA was extracted 7 d after transduction; and repair outcomes were assessed via amplicon sequencing (Methods). In accordance with our previous findings, the target sequences predicted to be cleaved in a blunt manner were mostly repaired as deletions, whereas the most common indel for staggered cuts was single-nucleotide insertions (Fig. 3l and Extended Data Fig. 5b). The insertion rate was significantly higher in the staggered pool compared to blunt (Extended Data Fig. 5c), and approximately 75% of all +1 indels were templated (Extended Data Fig. 5d). Collectively, these data indicate a strong association between the staggered Cas9 incisions with repair precision and predictability, highlighting the possibility of using predictions of Cas9 cleavage configurations for more precise and predictable genome editing.

## Genetic variation impacts Cas9 scission profile and editing outcome

**Extended Data Fig. 6:**
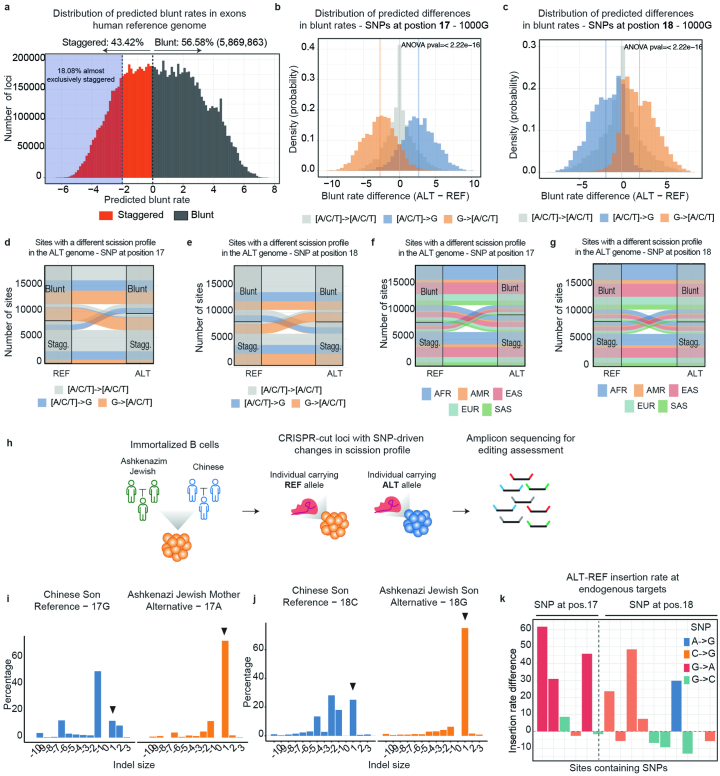
Predicting changes in scission profile driven by SNPs at key positions along the protospacer. **a**, Prediction of the blunt rate of every putative Cas9 target site found within exons in the human genome. Dashed lines mark thresholds at log2 rates of 0 (50% blunt DSBs, gray distribution; 50% staggered DSBs, orange distribution) and −2 (80% staggered DSBs, orange distribution). **b**, Distribution of predicted changes in blunt rates for SNPs found at position 17 for the 1000 G dataset. (two-sided ANOVA test comparing means, P-value < 2.2e-16). **c**, Distribution of predicted changes in blunt rates for SNPs found at position 18 for the 1000 G dataset. (two-sided ANOVA test comparing means, P-value < 2.2e-16) **d, e**, Sankey diagrams showing transitions between scission profile classes for SNPs found at positions 17 **(d)** and 18 **(e)**. The colors indicate genotype. Blunt threshold is log2 rate > 0, otherwise staggered. **f, g** Superpopulation-resolved Sankey diagrams showing predicted SNP-driven transitions between scission profile classes for positions 17 **(f)** and 18 **(g)**. AFR: African; AMR: American; EAS: East Asian; EUR: European; SAS: South Asian. **h**, Schematics of the experimental design for targeting the REF and ALT allele-containing GIAB donor B cells. **i**, Indel size distribution of the targeted locus containing an SNP at position 17 as shown in panel G. Indels of sizes between −10 and +3 were used for this analysis. Arrow heads indicate +1 indels. **j**, Indel size distribution of a locus containing an SNP at position 18 as shown in panel J. Indels of sizes between −10 and +3 were used for this analysis. Arrow heads indicate +1 indels. **k**, Difference in the insertion rate of target sites containing the indicated SNPs at position 17 or 18. Positive values indicate an increase in the insertion rate in the ALT allele, and negative values indicate a decrease in the insertion rate in ALT allele compared to REF. Source data

Given the strong dependency of Cas9 scission profile on the sequence context, we surveyed the entire coding human genome for putative Cas9 targets. We used our model to extrapolate the scission profile of every putative Cas9 target in human exons by predicting the blunt rate for over 10 million NGG-endowed sites. Our analysis indicated that 56.58% (5,869,863 of 10,374,276 sites) of putative Cas9 target sites are predicted to be cleaved predominantly in a blunt manner (log_2_ blunt rate > 0; equivalent to >50% blunt breaks) and 43.42% (4,504,413 of 10,374,276 sites) in a staggered configuration (log_2_ blunt rate < 0) (Extended Data Fig. 6a), with 18.08% of all target sites at human exons (1,875,201 of 10,374,276) to be cleaved in a highly staggered configuration (log_2_ blunt rate < −2; equivalent to >80% of staggered breaks) (Extended Data Fig. 6a). Because staggered Cas9-induced DNA breaks are strongly associated with precise and predictable single-nucleotide insertions, our findings suggest that predictable and precise genome editing might be favored by pre-selecting target sites that are predicted to be cleaved in a staggered configuration.

Single-nucleotide polymorphisms (SNPs) account for most human genetic variation^25^ and have the potential to affect Cas9 on-target and off-target activity^19,26–29^. However, the impact of human genetic variation on the scission profile of Cas9 has not yet been investigated. To understand how the genetic variation of an individual affects DNA scission by Cas9, we surveyed the 1000 Genomes Project (1000G) database for SNPs at positions 17 and 18 of putative Cas9 targets in exons, and we predicted blunt rates for Cas9 target sites in these different genomes using our machine learning model (Supplementary Table 5). As expected, based on the sequence determinants analysis (Fig. 3c), [A/C/T] > G substitutions at position 17 were associated with an increase in the blunt rate (more blunt breaks; 1,964 of 3,086 transitions), whereas G > [A/C/T] substitutions were associated with a decrease (more staggered breaks; 2,385 of 3,448 transitions) (Extended Data Fig. 6b,d,f). Conversely, at position 18, [A/C/T] > G substitutions were associated with more staggered breaks (1,973 of 2,859) and G > [A/C/T] with more blunt ones (1,569 of 2,679) (Extended Data Fig. 6c,e,g).

**Fig. 4:**
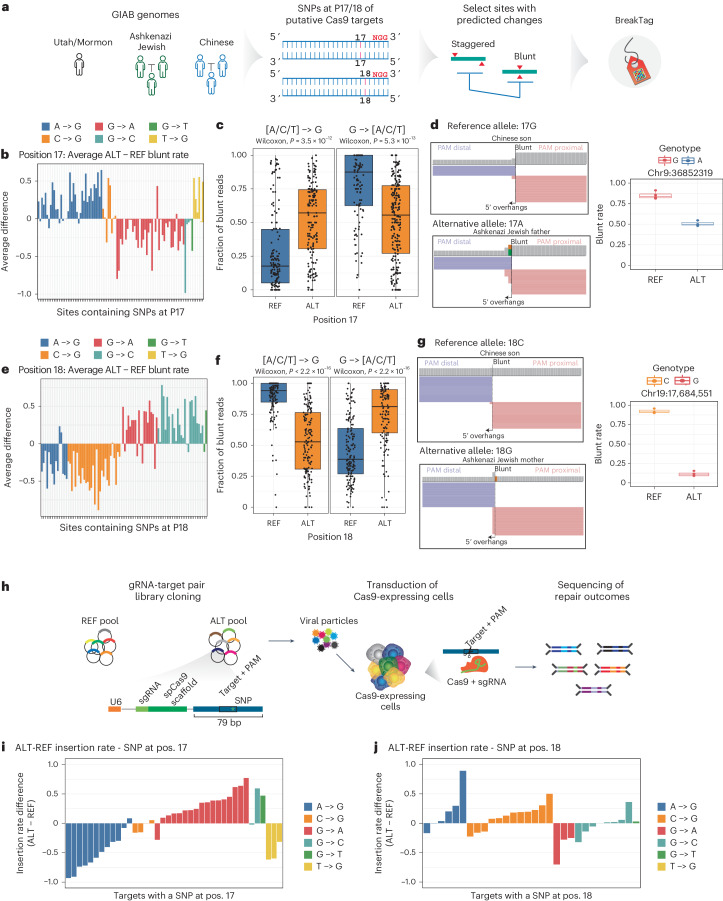
Human genetic variation influences Cas9 scission profile and indel outcome. **a**, Schematics of experimental design using SNP databases curated from the GIAB Consortium^30,31^. **b**, Average blunt rate difference between ALT and REF alleles with SNPs at position 17 of the protospacer, averaged by genotype. **c**, Fraction of blunt reads over the total number of sites with a SNP in position 17, comparing the reference (blue) and alternative (orange) alleles. Two-sided Wilcoxon test (*n* = 959 sites). Box plots show the lower quartile (Q1), median quartile (Q2) and upper quartile (Q3), with whiskers extending up to 1.5× the interquartile range (IQR = Q3−Q1) from the box edges. **d**, Left, representative IGV snapshot showing BreakTag reads of individuals harboring REF or ALT alleles. Right, the blunt rates for the REF and ALT genotypes for that locus (*n* = 7 genomes). Box plots show the lower quartile (Q1), median quartile (Q2) and upper quartile (Q3), with whiskers extending up to 1.5× the IQR (Q3−Q1) from the box edges. **e**, Difference in average blunt rate between ALT and REF alleles with SNPs at position 18, averaged by genotype. **f**, Fraction of blunt reads over the total number of sites with a SNP in position 18, comparing the reference (blue) and alternative (orange) alleles. Two-sided Wilcoxon test (*n* = 749 sites). Box plots show the lower quartile (Q1), median quartile (Q2) and upper quartile (Q3), with whiskers extending up to 1.5× the IQR (Q3−Q1) from the box edges. **g**, Left, a representative IGV snapshot showing BreakTag reads for REF and ALT alleles. Right, the blunt rates for the reference and alternative genotypes for that locus (*n* = 7 genomes). Box plots show the lower quartile (Q1), median quartile (Q2) and upper quartile (Q3), with whiskers extending up to 1.5× the IQR (Q3−Q1) from the box edges. **h**, Schematics of gRNA-target pair experiment for the ALT and REF pools. **i**, Difference in insertion rate of target sites with indicated SNPs at position 17, using targets with at least 100 mutated reads. **j**, Difference in insertion rate of target sites with indicated SNPs at position 18, using targets with at least 50 mutated reads. IGV, Integrative Genome Viewer; pos., position. Source data

To understand allele-specific changes in the Cas9 scission profile, we leveraged the genomes of seven individuals extensively characterized by the Genome-in-a-Bottle (GIAB) Consortium^30,31^. We first predicted the blunt rate of all loci containing a SNP at positions 17 (*n* = 394,330) or 18 (*n* = 395,368) among GIAB individuals using our machine learning model. Second, we predicted the effect of each base substitution in the Cas9 scission profile by calculating the difference between the predicted blunt rate for reference and alternative alleles. Based on our analysis, we selected 300 sites with a SNP at positions 17 or 18 and the highest predicted difference in blunt rate between the reference and alternative allele, with the goal of identifying SNP-driven changes in the Cas9 scission profile (Fig. 4a). Finally, we generated a HiPlex BreakTag dataset of 300 sites with SNPs targeting the reference or mutant allele (hereafter referred to as the ‘HiPlex3’ library) (Supplementary Table 6). We were able to confirm SNP-driven changes of scission profile predicted by our model in experimental observations. If a SNP was found at position 17 of the target site, an [A/T/C] > G substitution significantly increased the blunt rate, whereas G > [A/T/C] significantly reduced it (Fig. 4b–d). Analysis of position 18 revealed a strikingly opposite pattern, with [A/T/C] > G substitutions significantly decreasing the blunt rate and strongly associated with staggered DSBs, whereas G > [A/T/C] changes were significantly associated with blunt breaks (Fig. 4e–g).

Following our observation that Cas9 scission profile is a major determinant of repair outcome, we hypothesized that the SNP-driven changes in Cas9 cutting have the potential to change editing outcomes in an allele-specific manner. To test that, we leveraged our gRNA-target pair approach (Fig. 4h) to assess the indel outcomes of target sequences with a SNP at position 17 or 18 that displayed differences in scission profile in our BreakTag analysis (Fig. 4b–e). As expected by the strong association between the nucleotide type at positions 17 and 18 with the Cas9 scission profile, we observed changes in the editing outcome depending on the SNP type and position in the protospacer, with insertion rates changing according to the shift in the scission profile promoted by SNPs introducing or removing a G base at position 17 or 18 between the reference and alternative allele (Fig. 4i,j). We confirmed these findings by targeting endogenous loci containing a SNP at position 17 or 18 of the protospacer with known scission profiles in lymphoblastoid cell lines from B lymphocytes derived from GIAB donors, and we performed targeted ultra-deep sequencing (~10^6^×) (Extended Data Fig. 6h–k). As an example, a G > A substitution at position 17, which is associated with a higher proportion of staggered cuts (Fig. 4d), led to an increased frequency of +1 indels from 12% to 72% (Fisherʼs test, *P* < 2 × 10^−16^) (Extended Data Fig. 6i), whereas a C > G substitution at position 18, which also favors staggered Cas9 cuts (Fig. 4g), greatly increased the frequency of +1 indels from 25% to 75% (Fisherʼs test, *P* < 2 × 10^−16^) (Extended Data Fig. 6j).

Taken together, our data demonstrate that genetic variation directly impacts the Cas9 scission profile along with the editing outcome, highlighting the importance of implementing variant-aware analyses of the Cas9 scission profile for more predictable and precise genome editing.

## Engineered Cas9 variants with altered scission profiles

**Fig. 5:**
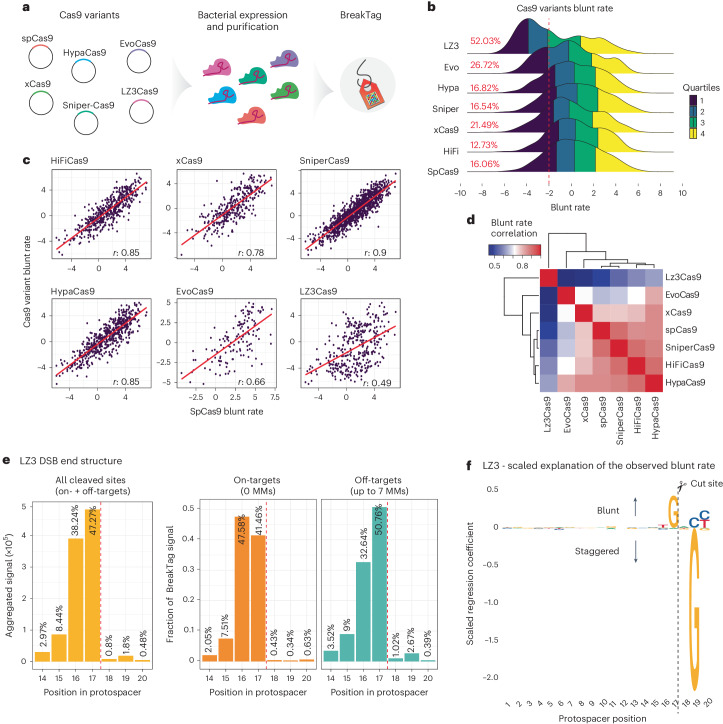
Cas9-engineered variants with modulated scission profiles. **a**, Schematic of the production of engineered Cas9 variants and characterization of scission profiles. **b**, Distribution of blunt rate for tested Cas9 variants for on-targets and off-targets. Colors show quartiles. The dashed line marks log_2_ rate of −2 (80% staggered DSBs). Sites with at least eight unique reads on the PAM-proximal side were used for the analysis. The percentage of sites with more than 80% staggered DSBs is shown. **c**, Blunt rate correlation between SpCas9 (*x* axis) and the tested variants (*y* axis). Each point is a cleaved site (on-target or off-target with at least eight unique reads on the PAM-proximal side of the break). **d**, Matrix depicting blunt rate correlation between the tested variants. **e**, Left, aggregated signal of different DSB end structures for on-targets/off-targets in the HiPlex1 library generated with the LZ3 nuclease. The fraction of blunt or staggered DSBs for on-targets (orange) and off-targets with up to seven mismatches (MM; green) are shown center and right, respectively. Position 17: blunt DSBs; 16–14: 5′ overhangs. The dotted line indicates the expected cut site for a blunt DSB. **f**, Observed LZ3 blunt rate explained by the sequence composition of the protospacer. Coefficients of a linear regression model fit to the nucleotide composition independently on each position of the protospacer are shown as letters scaled according to the importance of that nucleotide and position, as estimated by the XGBoost model. The dashed vertical line indicates a cut site for a blunt DSB. Source data

**Extended Data Fig. 7:**
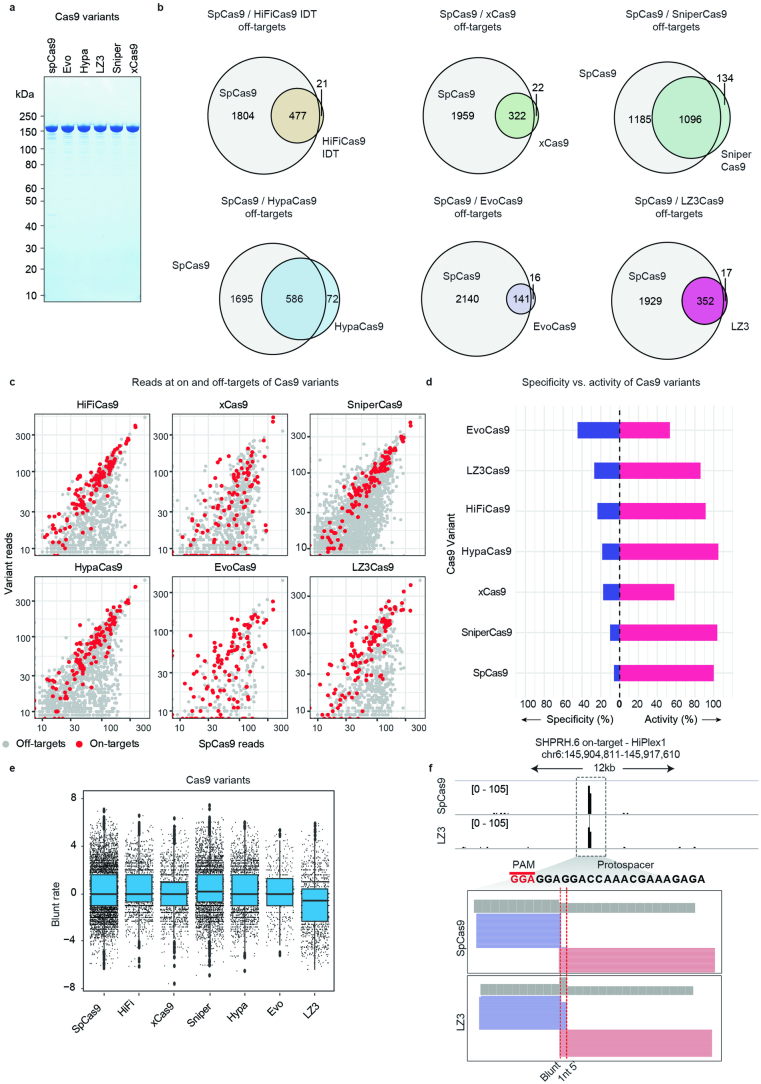
Cas9 variant specificity, activity and blunt rate analysis as measured by BreakTag. **a**, Coomassie Blue staining of recombinant Cas9 variants used here. **b**, Venn diagrams showing common cleaved sites mapped with BreakTag between SpCas9 and the tested Cas9 variant. Off-targets with at least 8 reads were used for this analysis. **c**, Reads at on and off-targets (up to 7 mismatches) for SpCas9 (x axis) and variants (y axis). Red dots indicate on-target signal and gray dots indicate off-targets. Off-targets with at least 8 reads were used for this analysis. **d**, Specificity (left direction) and activity (right direction) of tested Cas9 variants as calculated with BreakTag readout. Activity is reported in relation to SpCas9. **e**, Distribution of blunt rate for each Cas9 variant identified by BreakTag. Each point is a cleaved site (on-target or off-target). Blunt rate was calculated over 2 technical replicates. Boxes characterize the sample using the lower quartile (Q1), median (Q2) and upper quartile (Q3)—and the interquartile range (IQR = Q3 − Q1), and whiskers extend to the most extreme data point that is no more than 1.5 × IQR from the edge of the box. **f**, IGV snapshot showing an example of differential scission profile for the on-target sequence of SHPRH.6 sgRNA (HiPlex1 library). Source data

We demonstrated that the protospacer sequence is a major determinant of Cas9 cleavage pattern and the repair outcome, and therefore, pre-selecting target sequence composition can be leveraged for increased staggered cleavage favoring insertions. However, the sequence determinants dictating the Cas9 scission profile limit the number of targets that could be cleaved in a staggered manner, and, therefore, we set out to search for Cas9 variants with altered scission profiles. To this end, we characterized by BreakTag the scission profile of six previously described engineered variants with reduced off-target activity: HiFiCas9 (ref. ^32^), xCas9 (ref. ^33^), SniperCas9 (ref. ^34^), HypaCas9 (ref. ^35^), EvoCas9 (ref. ^36^) and LZ3Cas9 (ref. ^37^) (Fig. 5a and Extended Data Fig. 7a).

**Extended Data Fig. 8:**
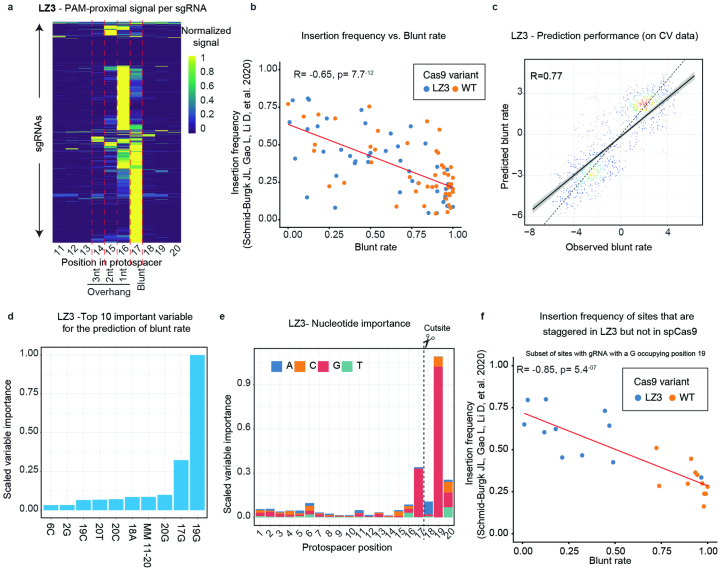
Characterization of the sequence determinants of the LZ3 flexible scission profile. **a**, Accumulation of reads mapped onto the PAM-proximal strand (scaled) along the protospacer over 4,543 sgRNAs of the HiPlex1 library generated with the LZ3 nuclease for all identified targets with an ‘NGG’ PAM. **b**, Correlation between insertion frequency and blunt rate calculated with BreakTag for 95 gRNAs for each Cas9 variant. **c**, Model performance evaluation using cross-validated (CV) data. This panel shows the correspondence between expected (predicted) and observed log2 ratio of reads indicating a blunt or a staggered cut. (Pearson correlation R = −0.65, P-value = 7.7e-12). (Pearson correlation R = 0.77). Dotted line represents perfect correlation (R = 1); error bands represent the 95% confidence interval around the linear model fit. **d**, Top ten most important variables for the prediction of LZ3 blunt rate. MM 11–20: mismatches in the seed part of the protospacer (positions 11–20). **e**, Top ten most important variables for the prediction of LZ3 blunt rate. MM 11–20: mismatches in the seed part of the protospacer (positions 11–20). **f**,Correlation between insertion frequency and blunt rate of the subset of 22 sites where a G occupied position 19 of the protospacer that are staggered when LZ3 was used but blunt when SpCas9 was used. (Pearson correlation R = −0.85, P-value = 5.4e-7). Source data

We performed BreakTag, targeting 150 genomic loci, and calculated the target specificity, the blunt rate and the overlapping off-targets for each variant. The variants displayed different levels of cleavage at on-targets and off-targets compared to SpCas9, with a marked reduction of overall cleavage for xCas9 and EvoCas9 (Extended Data Fig. 7b,c). Next, we calculated the relative ‘Activity’ (total on-target reads of variants normalized by total on-target reads of SpCas9) and ‘Specificity’ (proportion of off-target reads over on-target) of each variant, to investigate if there is a tradeoff between fidelity and overall cleavage activity. The variant EvoCas9 had the highest specificity score of all tested variants but displayed an approximately 47% reduction in activity compared to SpCas9 (Extended Data Fig. 7d). We observed no reduction of SniperCas9 and HypaCas9 on-target activity but a slight increase in specificity of approximately 4% and approximately 12%, respectively (Extended Data Fig. 7d). Strikingly, the variant LZ3 showed both a higher fidelity (Extended Data Fig. 7d) and a remarkable reduction of the blunt rate correlation versus SpCas9 (*r* = 0.49) (Fig. 5c,d and Extended Data Fig. 7e,f), along with a skewed distribution toward staggered breaks (Fig. 5b–e). We observed that approximately 48% of LZ3 DSB reads accumulated at position 17, reminiscent of blunt DSBs, whereas approximately 47% of breaks displayed 5′ overhangs (Fig. 5e). Most of the non-blunt breaks were 1-nt 5′ overhangs (38.24%), but 2-nt (8.44%) and 3-nt (2.97%) overhangs were also observed (Fig. 5e). Of note, the proportion of blunt to staggered breaks was gRNA dependent, indicating that, similar to SpCas9, LZ3’s scission profile is target sequence dependent (Extended Data Fig. 8a). In line with our findings, blunt rate and insertion frequency of SpCas9 and LZ3 were inversely correlated (*r* = −0.65, *P* = 7.7 × 10^−12^) (Extended Data Fig. 8b).

Given the marked reduction in correlation between the blunt rates of LZ3 and SpCas9 (Fig. 5c,d), we set out to further characterize the sequence determinants dictating LZ3’s scission profile. We applied a XGBoost regression model using the 2D one-hot-encoded representation of the correspondence between the 20 nt of the protospacer and guide sequences as predictors, together with the crRNA:DNA mismatches for BreakTag data on LZ3 (Extended Data Fig. 4a). The model achieved high performance as tested on cross-validated data (Extended Data Fig. 8c). We next investigated the most important variables and nucleotides along the protospacer for predicting the blunt rate, and, interestingly, a 19G target sequence had a high importance for predicting LZ3 target-specific blunt rate (Extended Data Fig. 8d,e). Similar to SpCas9, a 17G sequence was predictive of a blunt cut, but a 19G was highly predictive of a staggered DSB (Fig. 5f). To assess whether LZ3 could be used as an alternative of Cas9 to generate staggered breaks and produce insertions at target sites where Cas9 cleaves in blunt configuration, we investigated the insertion frequency at staggered DSBs generated by LZ3 but not by SpCas9. We indeed observed that LZ3 can generate higher insertion rates at staggered 19G sites compared to SpCas9 (Extended Data Fig. 8f), suggesting that a rational engineering of Cas9 variants might be a feasible strategy for introducing high-frequency insertion at target sequences where SpCas9 cleaves in a blunt manner.

## Leveraging scission profile for correction of pathogenic deletions

Given the strong link between scission profile and predictable insertions, we sought to test if a scission-based targeting strategy can be leveraged for correcting pathogenic single-nucleotide deletions. We reasoned that, by exploiting SpCas9 or engineered variant sequence determinants for staggered cleavage, single-nucleotide insertions can be favored, compensating frameshift mutations caused by a pathogenic deletion found in proximity to a PAM sequence. Furthermore, the predictability of insertions (Fig. 3i,j) would enable the recovery of the original protein sequence by exploiting codon degeneration.

**Fig. 6:**
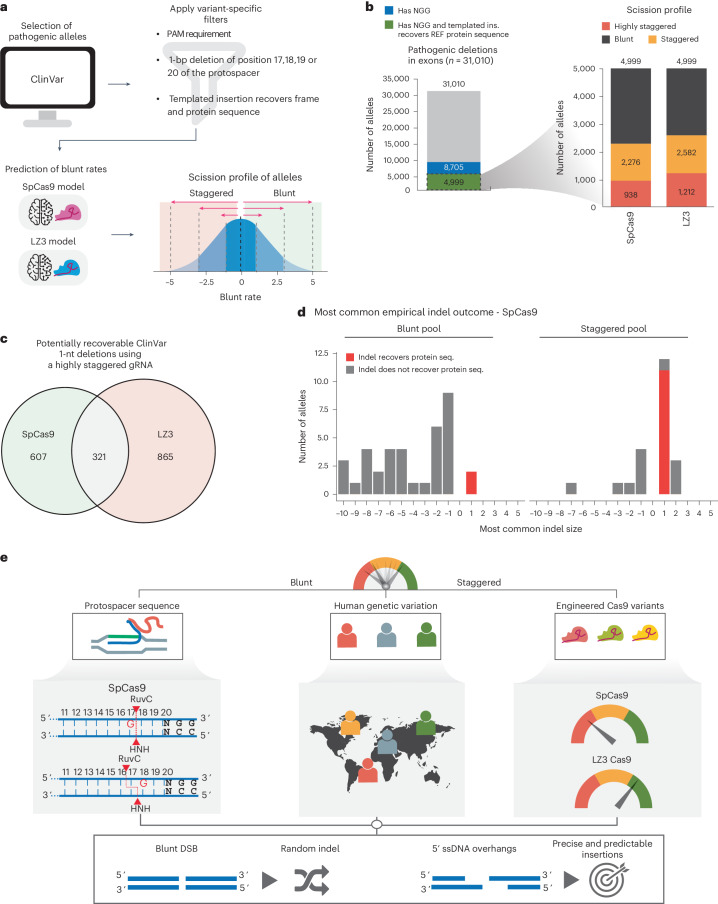
Cas9 variants expand the pool of pathogenic alleles amenable for correction. **a**, Schematics depicting the workflow for the prediction of scission-aware targeting of pathogenic deletions. **b**, Bar plot (left) shows the number of pathogenic deletions in exons that contain an NGG (blue) or that contain an NGG and a templated insertion recovers the reference protein sequence and frame (green). Horizontal bar plots (right) show the predicted scission profile of gRNAs targeting pathogenic deletions with LZ3 or SpCas9. Blunt indicates gRNAs with blunt rate > 0, staggered < 0 and highly staggered ≤ −2. **c**, Venn diagrams depicting the overlap between pathogenic alleles that are predicted to be cleaved in a highly staggered manner by LZ3 or SpCas9. **d**, Most common indel outcome for alleles in the blunt or staggered pool. **e**, A model of the determinants of Cas9 scission profile identified using BreakTag. The protospacer sequence, human genetic variation and engineering Cas9 variants can dictate Cas9 scission profile, which is strongly associated with precise and predictable genome editing. ins., insertion. Source data

**Extended Data Fig. 9:**
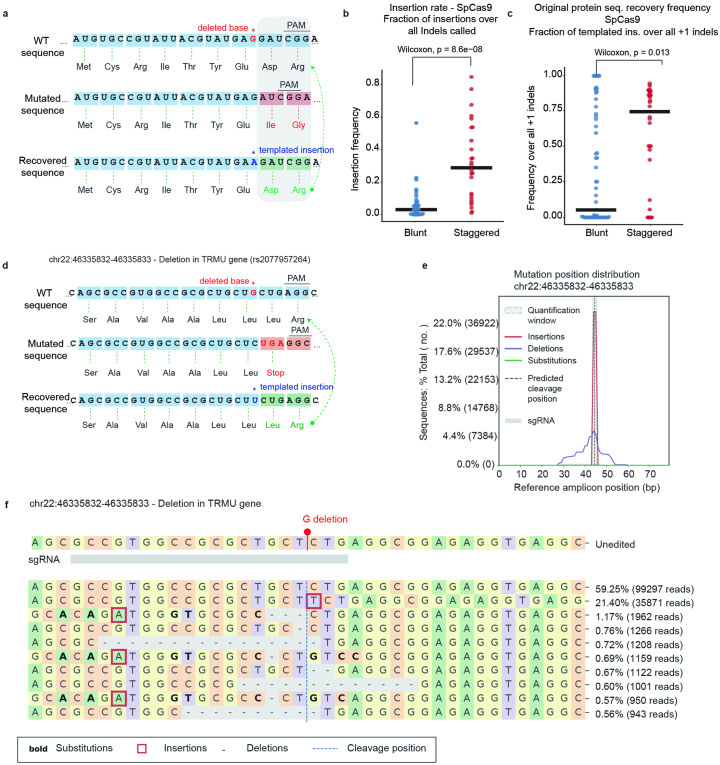
Investigation of indel outcomes at targeted pathogenic single-nucleotide deletions. **a**, Example of 1nt deletion generating a frameshift mutation, and a templated insertion rescuing the frame and original amino acid sequence. **b**, Insertion rate of pathogenic 1nt deletions predicted to be cleaved in a blunt or staggered manner. Horizontal lines represent the median values. A two-sided Wilcoxon test was performed to assess the significance of the differences observed between the mean signed ranks of the two conditions being compared (blunt vs. staggered P-value 8.6e-8; n = 145 independent Cas9-induced cutsites). **c**, Rate of original protein sequence recovery, as measured by the frequency of templated insertions (i.e, duplication of the base found at position 17 of the protospacer) over all +1 indels. Horizontal lines represent the median values. A two-sided Wilcoxon test was performed to assess the significance of the differences observed between the mean signed ranks of the two conditions being compared (blunt vs. staggered P-value 0.013; n = 145 independent Cas9-induced cutsites). **d**, Example of a pathogenic allele in the staggered pool. The 1nt deletion generates a stop codon in the TRMU gene, but the correct ORF is recovered upon templated +1 insertion. **e**, CRISPResso2^62^ output of the mutation outcome type distribution of the TRMU 1nt deletion depicted in Extended Data Fig. 9d**. f**, Table depicting the top 10 repair outcomes after targeting the 1nt deletion in the TRMU gene with SpCas9. Source data

To estimate how the acquired insights into the scission profiles of Cas9 variants can be leveraged for the correction of pathogenic deletions, we employed our models trained on HiPlex BreakTag data from SpCas9 or LZ3Cas9 to predict the scission profile of 1-nt pathogenic deletions included in the ClinVar database (Fig. 6a). Our goal was to assess the potential of inducing 1-nt templated insertions for correcting pathogenic deletions by restoring the frame and maintaining the original amino acid sequence, rescuing protein function (Extended Data Fig. 9a). In addition to SpCas9, we chose the LZ3Cas9 because it exhibits distinct scission profile sequence determinants that lead to higher insertion rates compared to SpCas9 at 19G loci (Figs. 3c and 5f and Extended Data Fig. 8f). From the 31,010 pathogenic single-nucleotide deletions found in exons cataloged in ClinVar, 8,705 were endowed by an NGG PAM and can be targeted by SpCas9 and LZ3 (Fig. 6b). A total of 4,999 NGG-endowed alleles were predicted to be restored if a templated insertion takes place, rescuing the healthy protein sequence (Fig. 6b). Next, we predicted the blunt rate of gRNAs targeting the candidate deletions for reframing and protein rescue using our model trained on SpCas9 and LZ3 (Supplementary Table 12). We observed that 2,276 alleles were predicted to be cut preferably staggered (blunt rate < 0) by SpCas9 and 2,582 by LZ3. From the staggered alleles, 938 were predicted to be cleaved in a highly staggered manner (blunt rate ≤ −2) by SpCas9 and 1,212 by LZ3, suggesting that templated insertions would be highly favored (Fig. 6b). From the highly staggered alleles, we observed that 321 were shared between both nucleases, but most were variant exclusive (607 for Cas9 and 865 for LZ3, in total 1,793 target sites), indicating that different sequence determinants expand the number of target sites that could be cleaved in a highly staggered manner for favoring templated insertions (Fig. 6c). We confirmed that pre-selection of target sites in which Cas9 induces staggered breaks compared to blunt increases the frequency of templated +1 insertions that could be used to rescue 39 pathogenic single-nucleotide deletions cataloged in ClinVar using the cellular assay used before (Fig. 3k). As anticipated, the insertion rate and the frequency of templated insertions over all +1 indels was significantly enriched in the subset of target candidates predicted to be cut highly staggered compared to highly blunt (*P* = 8.6 × 10^−8^) (Fig. 6d and Extended Data Fig. 9b,c), demonstrating, as proof of principle, that pre-selection of target sites in which Cas9 cuts staggered can be used to correct clinically relevant pathogenic deletions. Among those corrected deletions, a single-nucleotide deletion (ClinVar rs2077957264) in exon 1 creates a premature translational stop signal (p.Leu24*) in the *TRMU* gene, which has been reported to be associated with acute infantile liver failure^38^, and a gRNA targeting the deletion was predicted to be cut in a highly staggered manner (Extended Data Fig. 9d). Upon targeting this deletion, we observed that most indels were insertions (Extended Data Fig. 9e), with the vast majority being templated insertions (Extended Data Fig. 9f). The inserted base would recover the frame and the original amino acid sequence, disrupting the stop codon and recovering the original protein sequence (Extended Data Fig. 9d,f).

Taken together, our data suggest that predictable and precise gene editing is enhanced by controlling the Cas9 scission profile with three major determinants: sequence-governed rules for gRNA design, accounting for individual genetic variation and leveraging engineered Cas9 variants with differential scission profiles (Fig. 6e).

## Discussion

We developed and applied BreakTag to survey DSBs generated by Cas9 with over 3,500 sgRNAs in the human genome across different genomic backgrounds. Labeling free DSB ends preserves the directionality of sequencing reads and, coupled with an enzymatic treatment of ssDNA overhangs at the cut site, allows the systematic investigation of the scission profile of Cas9-mediated DNA breaks. BreakTag is a scalable methodology to profile the on-target and off-target Cas9 landscape along with a scission profile. Our work establishes BreakTag as a simple, quick and readily implemented high-throughput tool for assessing CRISPR safety for personalized genome editing, by testing gRNA specificity and scission on gDNA samples. We also report HiPlex BreakTag as a companion approach for targeting thousands of unique loci in a single experiment, enabling systematic analysis of the nuclease activity of CRISPR–Cas genome editors. By combining high-throughput in-house synthesis of sgRNA and targeting several genomic loci in the same pot, we generated robust datasets to probe the determinants of sgRNA specificity and Cas9 cleavage profile preference.

Off-target discovery tools can be grouped into different categories according to the nominating strategy. In cellulo tools, such as GUIDE-seq^22^ and TTISS-seq^37^, are highly sensitive methods that rely on the incorporation of double-stranded oligodeoxynucleotide (dsODN) tags at the cut site. Because the method relies on the co-delivery of the donor sequence with CRISPR to cells, toxicity has been reported in some models, such as induced pluripotent stem cells^39^, and delivery of the blunt dsODN requires optimization depending on the experimental model used. However, the excellent signal-to-noise ratio of the method poses a major advantage compared to biochemical assays, providing fewer ‘false positives’ (extensively reviewed in ref. ^40^). In vitro tools, such as SITE-seq^41^, DIGENOME-seq^42^, CIRCLE-seq^21^ and CHANGE-seq^19^, are sensitive approaches for nominating off-targets that rely on the sequencing of DSB ends generated by Cas9 in vitro and provide a list of sites that can be cleaved without chromatin and nuclear architecture present. However, none of the aforementioned methods allows the direct investigation of DSB end structure at scale, preventing a comprehensive scission profile investigation. BreakTag, in contrast, enables the nomination of off-targets for staggered-cleaving nucleases such as Cas12a and allows the parallel investigation of gRNA-specific scission profiles in multiple genomes in the same run, facilitating the study of genetic background-specific changes in scission profiles. One drawback is its relatively higher background compared to in cellulo methods, as it also sequences DSBs generated by intrinsic cell processes (for example, transcription and replication) and mechanical breaks during DNA extraction. These factors can potentially mask extremely low frequency off-targets falling within those regions.

Early studies identified a non-random repair outcome of Cas9-mediated breaks and a dependency on the target site sequence^6–9,13^. Evidence using molecular dynamics simulations suggested that binding of two catalytic Mg^2+^ ions at the RuvC domain could mediate flexible cleavage generating 1-bp 5′ overhangs, and biochemical evidence demonstrated that RuvC can cleave the non-target strand at different positions^3,15,16,18,43^. The flexible cleavage of RuvC was proposed to mediate precise and predictable insertions^8–12,14–17^, but the observed frequencies and determinants of staggered DSB ends were never investigated owing to the lack of tools for assessing scission profiles. Using BreakTag, we characterized, to our knowledge for the first time, the relative frequency of, and the factors that determine, the different types of Cas9-induced breaks. We observed that staggered ends represent approximately 35% of SpCas9 on-target and off-target DSBs, and we identified a strongly sgRNA-specific scission profile, highlighting that sequence context plays a role in the positioning of the RuvC domain. Our findings reveal a strong dependence of guanines in the RuvC cleavage site positioning. If guanine occupied position 17, the RuvC domain was more likely to cut between positions 17 and 18, generating a blunt DSB. Conversely, a guanine at position 18 shifted the RuvC cleavage site upstream of the HNH cut, generating staggered DSBs. Using a large matched dataset directly associating Cas9-induced scission profile with the repair outcome and a parallel assessment of repair outcomes of targets predicted to be cut in a blunt or staggered manner, we show that staggered DSBs generate predictable templated insertions with higher precision and that the frequency of templated insertions is increased by targeting sites with a guanine at position 18 for SpCas9. Because single-nucleotide insertions are the most common CRISPR–Cas9 repair outcome^6–11^, and are valuable for the correction of pathogenic alleles with single-base deletions or gene knockouts, our findings demonstrate that enhancing template-free precise and predictable genome editing is possible by selecting target sites with a staggered cleavage configuration. This is an achievable goal, as modeling the human genome revealed that approximately 18% of potential target sites found in exons are predicted to be cleaved by SpCas9 in a highly staggered configuration. The indel landscape is shaped by different DNA repair pathways influenced by the chromatin environment^44,45^, which might account for the slight deviation in sequence determinants of indels identified by computational predictors trained on repair outcome data^7–12^ compared to cleavage determinants identified by BreakTag.

Base editors and prime editors allow direct modification of the locus without relying on a DNA DSB, reducing the likelihood of misrepair that can lead to illegitimate chromosome joining^46^. However, base editors are limited to base conversions and cannot induce insertions^46^. Prime editors allow the formation of insertions, deletions and base conversions, but further development is necessary to increase editing efficiencies^47^. Although both prime and base editors bypass the need of a DNA DSB, recent evidence revealed the presence of genotoxic effects associated with this generation of editors, including deleterious deletions and translocations^48^. Cas9 scission profile-based pre-selection of gRNAs for precise insertions is limited to the correction of small deletions but still has a high translational potential as single-nucleotide deletions represent more than 31,000 of pathogenic variants in ClinVar (Fig. 6b,c).

Human genetic variation is ubiquitous and was shown to impact Cas9 on-target activity and the off-target landscape^19,26–29^. In the present study, we identified a central role for genetic variation in genome editing by CRISPR–Cas9 by demonstrating that the presence of SNPs at key positions along the protospacer modulate the indel outcome via changes in the Cas9 cleavage profile. More specifically, we directly demonstrate that SNPs found at positions 17 or 18 of the protospacer alter the SpCas9 scission profile, which dictates genome editing outcome. This notable finding has direct implications for the clinical use of CRISPR–Cas9. Altogether, our findings indicate that personalized genetic variation must be considered at the early stages of designing CRISPR–Cas9 targeting strategies. Furthermore, SNP-driven changes in Cas9 scission profile afford opportunities for precise allele-specific gene editing, and this places BreakTag as an experimental framework for predicting and identifying target sites susceptible to precise and desirable editing.

In a further step, we characterized the scission profile of several Cas9 variants and identified LZ3 as having a skewed distribution in favor of staggered DSBs. LZ3 has been identified as a Cas9 variant exhibiting a distinct insertional profile, with a preference of +1 indels at 19G loci^37^, further supporting our conclusion that an intrinsic link exists between scission profile and gene editing outcome. LZ3Cas9 contains four mutations—N690C (REC3), G915M (linker 2), N980K (RuvC) and T769J (linker 1)—that confer its higher specificity and/or altered scission profile. Interestingly, another study identified a G915F mutation in an engineered Cas9 variant with an altered scission profile^16^, indicating that interactions between the linker 2 (L2) domain and the non-target strand might promote a flexible scission. Of note, the residue Gly915 in L2 interacts with position 18 of the non-target strand^49^; a guanine at position 18 might change the interaction between the non-target strand and Cas9, displacing the RuvC cleavage site. SpCas9 demonstrated a higher incidence of blunt cuts at on-targets compared to off-targets, in line with previous findings on mismatched synthetic substrates for three gRNAs^18^. Interestingly, we show here that the LZ3 generates a higher proportion of staggered cuts at on-targets compared to off-targets, suggesting that the presence of mismatches can increase or decrease staggered cleavage in a variant-dependent manner. Taken together, the data-rich BreakTag workflow allows the assessment of variant fidelity, activity and determinants of nuclease scissions within a single assay, providing a platform for a fast, efficient and unbiased discovery of nuclease function.

Finally, we demonstrated how templated insertions can be explored for the correction of pathogenic single-nucleotide deletions. We leveraged flexible scission profile determinants of SpCas9 and LZ3 to predict pathogenic alleles amenable for precise corrective gene editing via predictable insertions. We envision that future development of engineered Cas9 variants with increased fidelity, alternate sequence determinants for staggered cleavage and decreased PAM requirements would expand the collection of sites amenable to precise gene editing.

In summary, we characterized the Cas9 endonuclease scission profile and established that the sequence of CRISPR–Cas9 target sites, human genetic variation and alternative Cas9 variants are three principal influencers of Cas9 cleavage pattern and, therefore, of gene editing outcomes. Our work illuminates the fundamental properties of Cas9 nuclease activity and lays the foundation for harnessing the flexible scission profile of Cas9 and engineered variants for precise, predictable and personalized genome editing.

## Methods

### Cell culture and genomic DNA extraction

Human osteosarcoma U2OS cells (American Type Culture Collection (ATCC)), human embryonic kidney cells (HEK293, ATCC) and HepG2 cells (a gift from Julian König’s laboratory) were cultured in DMEM (Gibco, 41965062) supplemented with 10% FBS (PAN-Biotech, P40-37500), 100 U ml^−1^ penicillin–streptomycin and 2 mM L-glutamine. K562-Cas9 cells (GeneCopoeia, SL552) were cultured in RPMI1640 medium (Gibco, 11875093) supplemented with 10% FBS (PAN-Biotech, P40-37500), 100 U ml^−1^ penicillin–streptomycin and 2 mM L-glutamine and kept under selection with hygromycin. HeLa Kyoto cells were infected with viral particles from LentiCas9-Blast (Addgene, 5292), and stable clones expressing Cas9 were maintained in DMEM supplemented with 10% FBS, 100 U ml^−1^ penicillin–streptomycin, 2 mM L-glutamine and 7 μg ml^−1^ blasticidin. Immortalized B cells from GIAB donors Chinese son (GM24631, Coriell), Chinese father (GM24694, Coriell), Chinese mother (GM24695, Coriell), Ashkenazi Jewish son (GM24385, Coriell) and Ashkenazi Jewish mother (GM24143, Coriell) were maintained in RPMI 1640 medium (Gibco, 11875093) supplemented with 15% FBS (PAN-Biotech, P40-37500), 100 U ml^−1^ penicillin–streptomycin and 2 mM L-glutamine. All cell lines were maintained in a humidified incubator at 37 °C supplemented with 5% CO_2_.

The gDNA of cells was extracted using a Qiagen Blood & Tissue Kit (Qiagen, 69506) following the manufacturer’s instructions and eluted in nuclease-free water.

gDNA of GIAB^30,31^ individuals was purchased from Coriell: female Utah/Mormon (NA12878), Ashkenazi Jewish son (NA24385), Ashkenazi Jewish father (NA24149), Ashkenazi Jewish mother (NA24143), Chinese son (NA24631), Chinese father (NA24694) and Chinese mother (NA24695).

### Expression and purification of homemade Tn5

Expression and purification of hyperactive Tn5 (E54K, L372P) were performed as described previously^50^ with the following modifications: Tn5 was expressed as an N-terminal His_6_–GST fusion followed by a 3C protease cleavage site. GSH affinity purification was used to capture the fusion protein, and it was subsequently cleaved using recombinant 3C protease.

### Tn5 loading and BreakTag linker preparation

Tn5-B adapter was prepared by mixing 100 µM Tn5ME-B and 100 µM Tn5MErev^51^ (Supplementary Table 7) resuspended in annealing buffer (50 mM NaCl, 40 mM Tris, pH 8) at a 1:1 ratio. The oligos were annealed in a thermocycler programmed as follows:

**Table:** 

Step	Temperature	Time
1	95 °C	5 min
2	65 °C	−0.1 °C s^−1^
3	65 °C	5 min
4	4 °C	−0.1 °C s^−1^
5	4 °C	Hold

Tn5 was loaded with pre-annealed Tn5-B adapter for 1 h at room temperature with agitation (300 r.p.m.) in a thermoshaker.

The BreakTag linker was prepared by combining 10 µM BreakTag_fwd and 10 µM BreakTag_rev oligos (Supplementary Table 7) in T4 polynucleotide kinase buffer (New England Biolabs (NEB), M0201S). The oligos were annealed in a thermocycler programmed as follows:

**Table:** 

Step	Temperature	Time
1	95 °C	5 min
2	Cool to 25 °C	−0.1 °C s^−1^
3	25 °C	Hold

### In vitro digestion of gDNA with Cas9 RNPs

RNPs were assembled by mixing Cas9 and sgRNA at equimolar ratios in NEB 3.1 buffer (NEB, B72030), followed by incubation at 37 °C for 10 min. For HiPlex BreakTag, pools were mixed with the nuclease at a 2:1 ratio. An input of 500 ng of gDNA was mixed with each RNP at a final concentration of 90 nM and incubated at 37 °C for 1 h in a thermocycler with the lid set at 37 °C. The reaction was terminated by adding RNase A (Thermo Fisher Scientific, 10753721) and proteinase K (NEB, P8107) at final concentrations of 0.8 µg µl^−1^ and 0.2 µg µl^−1^, respectively, at 37 °C for 20 min, followed by incubation at 55 °C for 20 min. Nuclease-digested gDNA was purified with DNA AMPure XP beads (1.2× volumes, Beckman Coulter, A63881).

### HiPlex sgRNA production

Sequences for HiPlex1 (ref. ^7^) and HiPlex2 (ref. ^10^) pools (Supplementary Table 1) were bioinformatically split into 10 pools. Each pool contained 150 gRNAs for HiPlex1 and 140 gRNAs for HiPlex2, modified as follows: the last nucleotide at the 5′ end of the gRNA sequence (position 20) was replaced with a G for efficient T7 transcription. A T7 promoter sequence 5′-GGATCCTAATACGACTCACTATAG-3′ was added at the 5′ end of the protospacer, and a SpCas9 scaffold sequence 5′-GTTTTAGAGCTAGAA-3′ was added at the 3′ end. The sequences were ordered as DNA oPools (Integrated DNA Technologies (IDT)) and reconstituted in nuclease-free water at 100 µM. In-house production of sgRNAs was performed using the HighYield T7 sgRNA Synthesis Kit (SpCas9) (Jena Bioscience, RNT-105) following the manufacturer’s instructions. In brief, each pool (1 µM) was used for an assembly PCR reaction using three primers: T7fwd_sRNA: 5′-GGATCCTAATACGACTCACTATAG-3′, T7rev_sgRNA: 5′-AAAAAAGCACCGACTCGG-3′ and SpCas9_scaffold: 5′-AAAAAAGCACCGACTCGGTGCCACTTTTTCAAGTTGATAACGGACTAGCCTTATTTTAACTTGCTATTTCTAGCTCTAAAAC-3′. To increase complexity and avoid PCR bias, we performed three separate PCR reactions for each pool, which were then combined before IVT. The expected size of the assembled DNA template was confirmed on an agarose gel and used directly for T7 IVT*.* Three IVT reactions per pool were performed for increased yield and were incubated for 90 min at 37 °C. IVT products were purified using 2× volumes of Agencourt RNAClean XP magnetic beads (Beckman Coulter, A66514) and resuspended in nuclease-free water. RNA concentration was estimated using Qubit RNA Broad Range (Invitrogen, Q10211).

### BreakTag procedure and sequencing

DNA DSB ends of nuclease-digested gDNA were repaired and 3′ adenylated using the NEBNext Ultra II End Repair/dA-Tailing Module (NEB, E7546) according to the manufacturer’s instructions with the following modification: the total volume of the reaction was halved by using half the volume of the reagents. Labeling of DSB ends by ligation with the BreakTag linker was performed using the NEBNext Ultra II Ligation Module (NEB, E7595) according to the manufacturer’s instructions with the following modifications: the total volume of the reaction was halved by using half the volume of the reagents, and the USER enzyme digestion step was omitted. The BreakTag linker was used at a final concentration of 50 nM per sample. Labeled DNA was size selected two times using 0.7× volumes of DNA AMPure XP beads (Beckman Coulter, A63987) and eluted in nuclease-free water. Tagmentation with in-house Tn5 was performed in freshly prepared 10 mM Tris-HCl (pH 7.5) buffer containing 10 mM MgCl_2_ and 25% *N*,*N*-dimethylformamide (DMF, Sigma-Aldrich, 227056). Tagmentation reactions were assembled using 100–200 ng of DSB-labeled DNA as input. Single-handle hyperactive Tn5 was used at a final concentration of 1.25 ng µl^−1^ per reaction. Tn5 was loaded with the Tn5ME-B oligonucleotide for 1 h at room temperature (Supplementary Table 7). The tagmentation mix was then incubated at 55 °C for 5 min in a pre-heated thermocycler followed by termination with 0.2% SDS at room temperature for 5 min. Libraries were amplified with NEBNext Ultra II Q5 Master Mix (NEB, M0544) in a thermocycler programmed as follows:

**Table:** 

Step	Temperature	Time	
1	72 °C	5 min	Gap-filling reaction
2	98 °C	30 s	
3	98 °C	10 s	
4	63 °C	30 s	14 loops (steps 3–5)
5	72 °C	60 s	
6	72 °C	5 min	
7	12 °C	Hold	

Amplified and barcoded samples were size selected by performing two consecutive 0.5× volume right-tail + 0.35× volume left-tail size (final volume 0.85x) selections using DNA AMPure XP beads (Beckman Coulter, A63987). Libraries were quantified using a Qubit dsDNA High Sensitivity Assay Kit or a sparQ Universal Library Quant Kit (QuantaBio, 95210-100), and fragment size distribution was assessed on a Bioanalyzer High Sensitivity DNA chip. Libraries were pooled and sequenced on a NextSeq 500/550 platform with NextSeq 500/550 High Output Kit v2 chemistry for SE 1 × 75 bp sequencing or NovaSeq PE 2 × 150 bp with a 15% PhiX spike-in.

### BreakTag data analysis with BreakInspectoR

Initial pre-processing was done in a Linux cluster using the BreakTag NGSpipe2go pipeline (https://github.com/roukoslab/breaktag). The pipeline processes raw reads as they are output by the sequencer and generates a BED file with coordinates containing DSBs. Raw reads (single-end or paired-end) were first scanned, and those not containing the expected 8-nt UMI followed by the 8-nt sample barcode in the 5′ end of read 1 were discarded. Valid reads were aligned to the human reference genome version hg38 downloaded from UCSC with timestamp of 15 January 2014, 21:14, using the ‘mem’ command in BWA (version 0.7.17-r1188)^52^ with a seed length of 19 and default scoring/penalty values for mismatches, gaps and read clipping. Reads mapped with a minimum quality score Q = 60 were retained to ensure that we worked only with uniquely mapping reads. A final de-duplication step was performed in which spatial consecutive reads mapping within a window of 30 nt, and their UMIs differing by up to two mismatches, were considered close PCR duplicates, and only one was kept. The resulting reads were aggregated per position and reported as a BED file.

Subsequent analysis was done using the BreakInspectoR package in R (https://github.com/roukoslab/breakinspectoR), which performs a guided search toward putative on-targets/off-targets. Starting from the previously generated BED files, BreakInspectoR identifies stacks of read ends near a PAM as candidate loci for containing a DSB, and it calculates a *P* value and a false discovery rate for each site identified, considering also the signal found in a non-targeted library. For HiPlex libraries, this process was sequentially repeated for all sgRNAs included in the pool. BreakInspectoR may identify ambiguous targets for sgRNAs in the pool that are separated by a Hamming distance of seven substitutions or less. Any ambiguous targets were removed from the list of all targets for a HiPlex library as necessary. The identification of sites required the function ‘breakinspectoR()’ to search for stacks of at least three read ends at a distance of 3 nt from an ‘NGG’ PAM, which is preceded by a protospacer sequence that differs by seven mismatches at most from the sgRNA sequence. Only breaks identified in standard chromosomes were retained. For the ‘PAM usage’ analysis (Fig. 1g), we called ‘breakinspectoR()’ with the same parameters but allowing any PAM (‘NNN’). RNA and DNA bulges in the off-targets nominated with BreakInspectoR were not excluded from the analysis.

### Blunt rate estimation

For each site identified by BreakInspectoR, we analyzed the scission profile using the ‘scission_profile_analysis()’ function. This function analyzes the signal in the PAM-proximal side and returns a table in the form of a ‘data.frame’ attached as metadata columns of a ‘GRanges’ object^53^. The table extends the coordinates of the original DSB with the signal found around the position at which the enzyme is expected to cut, a *P* value and a false discovery rate that assess the significance of the signal found outside the expected cut site compared to the non-target library and the classification of a site according to its preference for forming blunt or staggered breaks. We performed the analysis by using the function to look in a region between [−3, +3] nucleotides upstream/downstream of the expected cut site; for Cas9, this was 3 nt upstream (toward the 5′ end) from the PAM. To avoid sites that could mislead the analysis, we focused only on sites with an ‘NGG’ PAM, for which, in principle, expected cut sites are readily identified. Finally, from the table generated by ‘scission_profile_analysis()’, we could calculate the blunt rate for a site. We did this in two ways: (1) as a fraction of the signal found in the expected cut site (PAM 3 nt upstream—that is, position 17 of the protospacer) and the total amount of signal in the region [−3, +3] around the cut site and (2) as a log_2_ ratio of the signal in the expected cut site versus the signal in the region [−3, +3] around the cut site after excluding the signal in the cut site.

### Machine learning model for the prediction of blunt rates

We trained a machine learning model to predict scission profiles using the XGBoost flavor of the Gradient Boosting Machine algorithm implemented in the H2O.ai framework (Extended Data Fig. 4a). The software was installed in the Bioconductor R container release version 3.15 (ref. ^54^) (bioconductor/bioconductor_docker:RELEASE_3_15). We tuned the hyperparameters of the algorithm to use 1,000 trees of unlimited depth, DART as the booster algorithm^55^ and five folds for *K*-fold cross-validation with automatic fold assignment of instances.

Because the number and scission profiles of the identified targets differ greatly among sgRNA constructs, we used only a subset of the total identified targets as training instances. We selected only highly covered sites with at least 16 raw reads in the PAM-proximal side and accounted for specific biases. We limited the number of targets selected per sgRNA to 100 to avoid biases toward highly promiscuous sgRNA sequences and additionally sampled staggered targets with a probability *K*^*−1*^, where *K* is the ratio between the number of staggered (blunt reads < 20%) and blunt (blunt reads > 80%) targets for a specific sgRNA, to pick more from the pool of staggered targets and compensate for their under-representation in the total set of identified targets. This resulted in a final set of 18,759 ‘instances’ in the training set.

The ‘response’ variable to be predicted was the log_2_ ratio between the number of raw reads mapped in the PAM-proximal side exactly at position 17 of the protospacer (the expected cut site) and the sum of raw reads mapped in the PAM-proximal side found in positions 14–16 and 18–20 of the protospacer. A pseudocount was added to both the denominator and numerator of this fraction to avoid a division by 0.

We reflected in the ‘predictor’ variables both the on-target/off-target protospacer sequence and the actual gRNA sequence, along with the mismatches between the two. We performed one-hot encoding by constructing a 4 × 4 matrix for each of the 20 positions of the protospacer, each row representing one of the possible nucleotides (A, C, G, T) to occupy that position in the targeted protospacer, and in each column the same for the sgRNA sequence. The matrix was filled with ‘0’ with the exception of the cell representing the nucleotide in the protospacer (row) and the sgRNA (column) for that position, which would contain ‘1’. Each matrix was converted into a vector of length 16 by concatenating the column vectors, and, finally, the 20 vectors were concatenated into one large vector of length 320 with the final representation of the one-hot encoding. In addition, we included an additional predictor variable representing the number of mismatches between the targeted protospacer and the sgRNA sequence in the first 10 positions of the protospacer and a second variable representing the mismatches in the last 10 positions of the protospacer. In total, we used 322 variables to represent each training instance. Sequence motifs related to the scission profile were produced with the ggseqlogo package in R^56^.

### Selection of SNP-containing sites in GIAB genomes

We downloaded the VCF file containing the single-nucleotide variants (SNVs) called in GIAB^31^ (Supplementary Table 9). We filtered the files to retain SNPs only and retrieved the 20 bp of sequence context around those sites. We retained two subsets of 394,585 and 395,392 putative CRISPR–Cas9 target sites that contain an ‘NGG’ PAM preceded by a protospacer containing at positions 17 or 18 (respectively) a SNP found in at least one of the GIAB samples. We then used the reduced machine learning model, which uses only the last 10 positions of the protospacer, to predict the expected blunt rate of those putative target sites for the reference allele sequence targeted with an sgRNA matching the reference sequence and also for the mutated allele targeted with an sgRNA containing the mutation. The top 150 sites with the lowest blunt rates (75 in sense and 75 in antisense strands) and targets with the highest predicted changes were selected for HiPlex BreakTag sgRNA pool generation. For greater statistical power, we selected sites for which the alternative allele is found in three or four donors.

### GIAB SNP analysis

We used the ‘scission_profile_analysis()’ function in BreakInspectoR to obtain the scission profile of the 300 sites picked from the previously selected SNP-containing sites in GIAB genomes. We calculated the blunt rate as the fraction of the BreakTag signal in the expected cut site (position 17 of the protospacer) with respect to the total signal in the region [−3, +3] around the cut site, obtaining an approximation for the number of blunt breaks compared to the total number of breaks as captured by BreakTag. For the visualizations comparing the blunt rate and the genotype, we selected highly covered sites with at least 16 raw reads in the PAM-proximal side and reference and alternative genotype information in at least one sample for each genotype.

### 1000G database SNP analysis

The full set of biallelic SNVs and indels called by Lowy-Gallego et al.^57^ from phase three of the 1000 Genomes Project was downloaded from the EBI’s FTP server (http://ftp.1000genomes.ebi.ac.uk/vol1/ftp/data_collections/1000_genomes_project/release/20190312_biallelic_SNV_and_INDEL/ALL.wgs.shapeit2_integrated_snvindels_v2a.GRCh38.27022019.sites.vcf.gz) with the timestamp of 12 March 2019, 16:06. We further processed the file to keep only the SNPs that were called in at least 10% of the samples used in this call set (*n* = 5,248). The positions of the SNPs were cross-referenced with a table of all 11,431,163 putative CRISPR–Cas9 targets on exons annotated in the Ensembl version 98 database^58^ that have an NGG PAM. We shortlisted two subsets of 18,961 and 18,883 putative target sites with a SNP at positions 17 or 18 (respectively) of the protospacer sequence. We then used the reduced machine learning model, which uses only the last 10 positions of the protospacer, to predict the expected blunt rate of those putative target sites for the reference allele sequence targeted with an sgRNA matching the reference sequence and also for the mutated allele targeted with an sgRNA containing the mutation.

### Prediction of blunt rates of gRNAs targeting pathogenic deletions

The full set of variants annotated in ClinVar as of April 2023, comprising a total of 2,122,310 variants, was downloaded from the National Institutes of Health FTP server (https://ftp.ncbi.nih.gov/pub/clinvar/vcf_GRCh38/clinvar.vcf.gz). Only variants that were 1-nt deletions, located in standard chromosomes, overlapping an exon annotated in TxDb.Hsapiens.UCSC.hg38.knownGene (data package made from resources at UCSC on 16:50:30 + 0000, Thursday, 7 April 2022) and annotated in ClinVar as ‘Pathogenic’ or ‘Likely_pathogenic’, were considered (31,010 variants). We focused on a subset of 8,705 deletions that had an NGG motif directly adjacent to them in either strand and up to 4 nt upstream. Those sites were candidates for being cut by Cas9 in a staggered manner, which could potentially induce a templated +1 insertion as the repair outcome, correcting the frameshift in the pathogenic allele and potentially recovering the original protein sequence. We calculated that a total of 4,999 of those deletions would recover the original protein sequence with a templated +1 insertion. Next, we designed ‘in silico’ the gRNA sequences that would target the regions containing the deletions, and we estimated the blunt rate using the previously described XGBoost models for SpCas9 and LZ3 trained with the HiPlex library. Those sites predicted to be cut in a highly staggered manner (log_2_ blunt rate < −2) in which a templated insertion would recover the original protein were finally reported as pathogenic variants being potentially treated with a CRISPR–Cas9 therapy.

### Construction of gRNA-target pair lentiviral libraries

Using our XGBoost models for SpCas9, we predicted the blunt rate of human genome sites and selected 150 sites predicted to be cut mostly blunt and 150 sites predicted to be cut mostly staggered. For the ‘ALT’ and ‘REF’ libraries, all gRNAs used in the HiPlex3 dataset were used. The cloning strategy of gRNA-target pair lentiviral libraries was adapted from Allen et al.^10^. In brief, a scaffoldless lentiviral expression vector, pKLV2-U6(BbsI)-PKGpuro2ABFP-W, was generated by removing the improved gRNA SpCas9 scaffold from pKLV2-U6gRNA5(BbsI)-PKGpuro2ABFP-W34 (gift from Kosuke Yusa, Addgene plasmid no. 67974). The deletion was generated by amplifying two fragments encompassing the 5′ end of the AmpR cassette to U6 promoter and PGK promoter of the 3′ end of the AmpR cassette, followed by Gibson assembly. The empty vector was transformed into Stabl3 chemically competent cells; single colonies were picked; and scaffold deletion was confirmed via Sanger sequencing.

For the library cloning step, we generated a 170-nt oligonucleotide pool (IDT) encoding the gRNA and a portion of the allele sequence containing 79 nucleotides with the target sequence + PAM in the center for the four individual libraries (Extended Data Fig. 5a). The oligonucleotide was amplified with primers compatible with the scaffold used, and a Gibson assembly was used to fuse the amplified pool to a 193-nt Ultramer duplex (IDT) encoding the improved version of the gRNA scaffold and a spacer sequence^10^. Three separated Gibson assembly reactions were performed per pool at a 1:1 molar ratio, followed by an incubation for 1 h at 50 °C, and subsequently pooled for column-based purification (Monarch PCR & DNA Cleanup Kit, NEB, T1030S), and removal of linear DNA was achieved by treating the samples with Plasmid-Safe ATP-Dependent DNAse (Epicentre). The intermediate circular insert and scaffoldless vector were linearized with a FastDigest BpiI (IIs class) kit (Thermo Fisher Scientific, FD1014) for 30 min and ligated in triplicates per pool (T4 DNA ligase, NEB, M0202). The replicates were pooled and transformed in Stabl3 chemically competent cells.

### Transduction of gRNA-target lentiviral pools

For lentiviral packaging of gRNA-target libraries, the gRNA-target libraries were independently co-transfected with the two packaging plasmids, and the supernatants were pooled and concentrated 50–100-fold. Packaging and transduction were performed as described previously^59^. In brief, we produced the viruses by co-transfection of 293T cells with each of the four library pools and two helper plasmids, psPAx2 and pMD2.g, encoding the VSV-G envelope and the lentiviral gag-pol genes, respectively. We harvested the lentiviral vector-containing supernatant twice, at approximately 42 h and 66 h after transfection, and concentrated it by using Lenti-X Concentrator (Takara, 631232). We plated 300,000 cells in a well of a six-well plate and transduced with the vector supernatants and 4 μg ml^−1^ polybrene in a total volume of 2 ml. After 48 h, the transduced cells were removed from the six-well plate, and one fifth of the cells were tested for BFP expression by flow cytometry (BD Canto), whereas the rest were plated in 10-cm^2^ tissue culture dishes for selection with puromycin (1 μg ml^−1^). Cells were kept under puromycin selection for 5 d. On the last day, cells were collected and tested for BFP expression, and gDNA was isolated using the Qiagen Blood & Tissue Kit (Qiagen, 69506).

### gRNA-target pair amplicon sequencing library preparation

The region containing the gRNA sequence and 79-nt portion of the allele was amplified using the Fwd_pool and Rev_pool primers (Supplementary Table 13) with NEBNext Ultra II Q5 Master Mix (NEB, M0544) with the following program: 98 °C for 60 s, 24 loops of 98 °C for 10 s and 72 °C for 30 s, followed by a final extension at 72 °C for 2 min. The PCR product was purified using 0.9× volumes of DNA AMPure XP beads (Beckman Coulter, A63987) and eluted in nuclease-free water. The cleanup product was used for a second PCR round with indexed primers (Supplementary Table 13) with the following conditions: 98 °C for 60 s, 13 loops of 98 °C for 10 s, 67 °C for 10 s and 72 °C for 20 s, followed by a final extension at 72 °C for 2 min. The indexed libraries were pooled, and the band corresponding to the amplicon size (464 bp) was excised from a 2% agarose gel, purified and sequenced in paired-end mode (2 × 150 bp) in a NextSeq 2000 sequencer with 40% PhiX spike-in.

### Analysis of gRNA-target repair outcomes

The first read in pair was used solely to estimate the abundance of each gRNA, as it reads into the gRNA portion of the construct. The second pair that reads into the target sequence was reverse complemented with the fastx_toolkit (http://hannonlab.cshl.edu/fastx_toolkit) and stripped from the first 57 bases and kept only the immediate 79 nt using Trimmotatic^60^ with options SE HEADCROP:57 CROP:79, which would keep only the 79-nt-long portion of the read containing the actual amplicon of the targeted sequence. Processed reads from technical replicates were merged in a single FASTQ file, and indels were called using CRISPResso2 (ref. ^61^) in pooled mode (CRISPRessoPooled), restricting the analysis to regions with at least 100 aligned reads and ignoring substitutions other than indels. gRNAs with detected activity in wild-type (WT) cells not expressing Cas9 that had been reported in the CRISPResso2 analysis with at least 100 edited reads were excluded from the analysis. For the rest, we extracted from the CRISPResso2 analysis output the length of the indel, the frequency of the most common +1 insertion over all edited sequences and the inserted nucleotide.

### Nucleofection of RNP complexes into lymphoblastoid cells

For the preparation of RNP complexes, sgRNAs targeting SNP-containing loci (Supplementary Table 8) were generated in-house using the HighYield T7 sgRNA Synthesis Kit (SpCas9) (Jena Bioscience, RNT-105). Two hundred picomolar sgRNA was mixed with 100 pM Alt-R S.p. Cas9-GFP V3 (IDT, 10008100) and incubated at room temperature for 10 min. A total of 5 × 10^5^ cells per reaction were resuspended in SF Cell Line 4D-Nucleofector solution (Lonza, V4XC-2032) and nucleofected in a 4D-NucleoFector system using the pulse code DN-100. Nucleofected cells were transferred to a plate containing culture medium and kept in a humidified incubator at 37 °C supplemented with 5% CO_2_ for 3 d before gDNA was extracted for indel analysis.

### Amplicon sequencing and editing analysis using CRISPResso2

The gDNA of lymphoblastoid cells nucleofected with RNPs was extracted 3 d after CRISPR delivery. Approximately 100 ng of gDNA from each sample was used for locus amplification using the primers listed in Supplementary Table 8. Amplicon libraries were generated as described previously^62^ with the following modifications: a first round of amplification using NEBNext Ultra II Q5 Master Mix (M0544) was performed with 33 cycles. The amplified DNA was purified using a 1× volume of DNA AMPure XP beads (Beckman Coulter, A63987), and the entire purified product was used for a second round of PCR with primers containing p5 and p7 sequences for Illumina sequencing (Supplementary Table 8). Amplicons were pooled and sequenced in a MiniSeq sequencer in single-read mode and 150 cycles.

Indel analysis was performed in a local Linux cluster using CRISPresso2 in pooled format^61^ using the following parameters: –*amplicon_min_alignment_score 50–quantification_window_size 10–quantification_window_center -3–exclude_bp_from_left 0–exclude_bp_from_right 0–ignore_substitutions–plot_window_size 20–min_frequency_alleles_around_cut_to_plot 0*.

### Cas9 variant cloning, expression and purification

The pET-Cas9-NLS-6×His expression vectors for Cas9 variants were generated by using Gibson assembly. As a PCR template for the expression vector backbone, pET WT Cas9-NLS-6×His was used^63^ (Addgene plasmid no. 62933). The PCR templates for the Cas9 variants were pX165-LZ3 Cas9 (Addgene plasmid no. 140561), pX165-evoCas9 (Addgene plasmid no. 140569), pX165-xCas9 (Addgene plasmid no. 140568), pX165-HypaCas9 (Addgene plasmid no. 140567) and pX165-SniperCas9 (Addgene plasmid no. 140560).

The pET expression vectors were transformed into *Escherichia coli* BL21 (DE3) CodonPlus (Agilent) and grown at 37 °C and 140 r.p.m. until an optical density at 600 nm (OD_600_) value of 0.5 was achieved. Cultures were cooled to 18 °C on ice, and protein expression was induced using IPTG at a final concentration of 0.5 mM and incubated for a further 21 h at 18 °C and 140 r.p.m. Cells were harvested by centrifugation (4,000*g*, 15 min), resuspended in ice-cold lysis buffer (30 mM Tris-HCl, 500 mM NaCl, 10 mM imidazole, 1 mM MgCl_2_, 1 mM TCEP, 5% glycerol, 1× complete protease inhibitor, 100 U ml^−1^ benzonase, pH 8.0) and lysed by high-pressure homogenization at 28 kpsi (Constant Systems CF1 Cell Disruptor). Cells were cleared by centrifugation (40,000*g*, 30 min, 4 °C), and the cleared lysate was applied to a HisTrap FF 5-ml column (Cytiva), using an automated chromatography system (Bio-Rad, NGC Quest Plus; used for all chromatography steps). The column was washed with 20 CV wash buffer (30 mM Tris-HCl, 500 mM NaCl, 10 mM imidazole, 5% glycerol), and the Cas9 variants were eluted from the Ni–NTA column by applying a linear gradient of 10–500 mM imidazole (containing 30 mM Tris-HCl, 500 mM NaCl, 5% glycerol). The eluted proteins were diluted 1:10 in a low-salt buffer (25 mM Na–HEPES, pH 7.2, 100 mM NaCl, 5% glycerol), applied to a HiTrap Heparin 5-ml column (Cytiva) and eluted by applying a linear NaCl gradient from 100 mM to 1,000 mM. Elution fractions containing the Cas9 variants were pooled and concentrated using Amicon Ultra-15 spin concentrators (Merck). Concentrated proteins were applied to a gel filtration column (Superdex 200 16/60 pg, Cytica, 40 mM Na–HEPES, pH 7.4, 400 mM NaCl, 10% glycerol). Peak fractions containing the Cas9 variants were pooled, concentrated to 6.4 g L^−1^ and diluted 1:2 with 86% glycerol to a final concentration of 3.2 g L^−1^ (20 µM). HiFiCas9 was purchased from IDT (no. 1081060).

### Reporting summary

Further information on research design is available in the Nature Portfolio Reporting Summary linked to this article.

## Online content

Any methods, additional references, Nature Portfolio reporting summaries, source data, extended data, supplementary information, acknowledgements, peer review information; details of author contributions and competing interests; and statements of data and code availability are available at 10.1038/s41587-024-02238-8.

## Supplementary information

Supplementary Note

Reporting Summary

Supplementary Tables 1–13

## Source data

Source Data Fig. 1 Statistical Source Data

Source Data Fig. 2 Statistical Source Data

Source Data Fig. 3 Statistical Source Data

Source Data Fig. 4 Statistical Source Data

Source Data Fig. 5 Statistical Source Data

Source Data Fig. 6 Statistical Source Data

Source Data Extended Data Fig. 1 Statistical Source Data

Source Data Extended Data Fig. 2 Statistical Source Data

Source Data Extended Data Fig. 3 Statistical Source Data

Source Data Extended Data Fig. 4 Statistical Source Data

Source Data Extended Data Fig. 5 Statistical Source Data

Source Data Extended Data Fig. 6 Statistical Source Data

Source Data Extended Data Fig. 7 Statistical Source Data

Source Data Extended Data Fig. 8 Statistical Source Data

Source Data Extended Data Fig. 9 Statistical Source Data

## Data Availability

All genomics data produced in this study have been deposited in the Gene Expression Omnibus under accession number GSE223772 (ref. ^64^). Source data are provided with this paper.

## Code availability

The BreakInspectoR pipeline and relevant bioinformatics pipelines used in this study can be found at https://github.com/roukoslab/breaktag (ref. ^65^) and at https://github.com/roukoslab/breakinspectoR (ref. ^66^).
