# Supplementary material for: Linking CRISPR–Cas9 double-strand break profiles to gene editing precision with BreakTag
Source: Nat Biotechnol. 2024 May 13;43(4):608–22. doi: 10.1038/s41587-024-02238-8 (PMC11994453; doi:10.1038/s41587-024-02238-8)
Supplement: Supplementary file 1 — Supplementary Note [file 41587_2024_2238_MOESM1_ESM.pdf]

nature biotechnology

Article https://doi.org/10.1038/s41587-024-02238-8

Linking CRISPR–Cas9 double-strand break profiles to gene editing precision with BreakTag

In the format provided by the authors and unedited

Supplementary note 1:

BreakTag DNA double-strand break amplification strategy

BreakTag is a highly scalable four-step protocol that maps free DSB ends in gDNA digested *in vitro* to RNPs. Ready-to-sequence libraries are achieved in less than 6 hours with minimal hands-on time. The method is performed in multi-well plates with the use of a multichannel pipette, and automation is simple. The procedure starts with a blunting step, in which 5' overhangs are filled-in and 3' overhangs are resected followed by A-tailing where a single adenine is added to the 3' end of the DSB prior to labeling. Processed ends are then ligated with a customized BreakTag linker. The linker contains a PCR handle, the sequencing primer binding site (mosaic end, ME), a unique molecular identifier (UMI) for removal of PCR duplicates, and a sample barcode. The sample barcode, which is embedded in the linker, allows an extra layer of barcoding and increases the throughput, such that samples can be pooled and further processed in the same tube if necessary. After ligation with the BreakTag linker, the gDNA is tagmented with a single-handle Tn5 containing a second PCR handle, which cuts the DNA randomly and inserts an adapter into the 5' end of the fragment [51]. Ligation of DSB ends with the BreakTag linker followed by tagmentation with single-handle Tn5 generates two populations of fragments, one termed "homotagged", in which both ends of the fragment contain the same sequence added during tagmentation, and a second "heterotagged" population, in which fragments contain the BreakTag linker at one end and the tagmentation linker at the other. The latter are amenable for exponential amplification as they contain two distinct PCR handles for primers that introduce functional p5 and p7 sequences. Homotagged fragments <1kb are not exponentially amplified and do not cluster during sequencing with Illumina sequencers (Fig. 1a).
