# Supplementary material for: Linking CRISPR–Cas9 double-strand break profiles to gene editing precision with BreakTag
Source: Nat Biotechnol. 2024 May 13;43(4):608–22. doi: 10.1038/s41587-024-02238-8 (PMC11994453; doi:10.1038/s41587-024-02238-8)
Supplement: Supplementary file 2 — Reporting Summary [file 41587_2024_2238_MOESM2_ESM.pdf]

nature portfolio

Corresponding author(s): Vassilis Roukos

Last updated by author(s): Apr 8, 2024

Reporting Summary

Nature Portfolio wishes to improve the reproducibility of the work that we publish. This form provides structure for consistency and transparency in reporting. For further information on Nature Portfolio policies, see our Editorial Policies and the Editorial Policy Checklist.

Statistics

For all statistical analyses, confirm that the following items are present in the figure legend, table legend, main text, or Methods section.

n/a	Confirmed	
☐	☒	The exact sample size (*n*) for each experimental group/condition, given as a discrete number and unit of measurement
☐	☒	A statement on whether measurements were taken from distinct samples or whether the same sample was measured repeatedly
☐	☒	The statistical test(s) used AND whether they are one- or two-sided<br>*Only common tests should be described solely by name; describe more complex techniques in the Methods section.*
☐	☒	A description of all covariates tested
☐	☒	A description of any assumptions or corrections, such as tests of normality and adjustment for multiple comparisons
☐	☒	A full description of the statistical parameters including central tendency (e.g. means) or other basic estimates (e.g. regression coefficient) AND variation (e.g. standard deviation) or associated estimates of uncertainty (e.g. confidence intervals)
☐	☒	For null hypothesis testing, the test statistic (e.g. *F*, *t*, *r*) with confidence intervals, effect sizes, degrees of freedom and *P* value noted<br>*Give P values as exact values whenever suitable.*
☒	☐	For Bayesian analysis, information on the choice of priors and Markov chain Monte Carlo settings
☒	☐	For hierarchical and complex designs, identification of the appropriate level for tests and full reporting of outcomes
☐	☒	Estimates of effect sizes (e.g. Cohen's *d*, Pearson's *r*), indicating how they were calculated

Our web collection on statistics for biologists contains articles on many of the points above.

Software and code

Policy information about availability of computer code

Data collection	Fastq sequencing data obtained with bcl2fastq v2.19.l.403 (Illumina, Inc)
Data analysis	Sequencing reads were aligned with bwa 0.7.17-r1188; pre and post-processing of reads with custom scripts written and executed in Perl v5.34.0 and Python 3.10.4; statistical analysis was performed usign R version 4.2.0 (2022-04-22) and Bioconductor 3.16; BreakTag data analysis was performed with breakinspectoR 0.1.0; Amplicon sequencing data was analyzed with CRISPResso2 v2.2.12

For manuscripts utilizing custom algorithms or software that are central to the research but not yet described in published literature, software must be made available to editors and reviewers. We strongly encourage code deposition in a community repository (e.g. GitHub). See the Nature Portfolio guidelines for submitting code & software for further information.

Data

Policy information about availability of data

All manuscripts must include a data availability statement. This statement should provide the following information, where applicable:

- Accession codes, unique identifiers, or web links for publicly available datasets
- A description of any restrictions on data availability
- For clinical datasets or third party data, please ensure that the statement adheres to our policy

All genomics data produced in this study have been deposited in GEO under accession number GSE223772 (https://www.ncbi.nlm.nih.gov/geo/query/acc.cgi?acc=GSE223772).The BreakInspectoR pipeline and relevant software used in this study can be found at https://github.com/roukoslab/breaktag and at https://

github.com/roukoslab/breakinspectoR.

Human research participants

Policy information about studies involving human research participants and Sex and Gender in Research.

Reporting on sex and gender	N/A
Population characteristics	N/A
Recruitment	N/A
Ethics oversight	N/A

Note that full information on the approval of the study protocol must also be provided in the manuscript.

Field-specific reporting

Please select the one below that is the best fit for your research. If you are not sure, read the appropriate sections before making your selection.

☒ Life sciences ☐ Behavioural & social sciences ☐ Ecological, evolutionary & environmental sciences

For a reference copy of the document with all sections, see nature.com/documents/nr-reporting-summary-flat.pdf

Life sciences study design

All studies must disclose on these points even when the disclosure is negative.

Sample size	Following the guidelines for Chip-seq experiments of the ENCODE Consortium, at least 2 replicates were prepared and sequenced for BreakTag experiments, since BreakTag relies on the same methods as Chip-seq.
Data exclusions	No data were excluded.
Replication	BreakTag experiments were carried in at least 2 replicates. gRNA-target experiments were replicated independently at least once. All attempts at replication were successful. For BreakTag samples, consistency was assessed by calculating the Pearson correlation coefficient between replicates of the signal in the identified double-strand breaks.
Randomization	No randomization was performed in our study as appropriate control samples were used to establish baseline activities. Randomization is not relevant to our study.
Blinding	No blinding was performed in our study as appropriate control samples were used to establish baseline activities. Blinding is not relevant to our study.

Reporting for specific materials, systems and methods

We require information from authors about some types of materials, experimental systems and methods used in many studies. Here, indicate whether each material, system or method listed is relevant to your study. If you are not sure if a list item applies to your research, read the appropriate section before selecting a response.

Materials & experimental systems

n/a	Involved in the study	
☒	☐	Antibodies
☐	☒	Eukaryotic cell lines
☒	☐	Palaeontology and archaeology
☒	☐	Animals and other organisms
☒	☐	Clinical data
☒	☐	Dual use research of concern

Methods

n/a	Involved in the study	
☒	☐	ChIP-seq
☒	☐	Flow cytometry
☒	☐	MRI-based neuroimaging

Eukaryotic cell lines

Policy information about cell lines and Sex and Gender in Research

Cell line source(s)	Human osteosarcoma U2OS cells (HTB-96 ATCC), human embryonic kidney cells (HEK293, CRL-1573 ATCC) and Hep-G2 cells (ACC 180 DSMZ). K562-Cas9 cells (Genecopoeia, SL552). Immortalized B cells from Genome-in-a-bottle donors Chinese son (GM24631, Coriell), Chinese father (GM24694, Coriell), Chinese Mother (GM24695, Coriell), Ashkenazi Jewish son (GM24385, Coriell), and Ashkenazi Jewish mother (GM24143, Coriell) were all obtained from Coriell. HeLa-Cas9 cells were generated in this study from Hela-Kyoto cells (CCL-2, ATCC).
Authentication	None of the cell lines used was authenticated.
Mycoplasma contamination	All cells were tested negative for mycoplasma.
Commonly misidentified lines (See ICLAC register)	No commonly misidentified cell lines were used in this study.
